# Simulation of Ultrafast Excited-State Dynamics in Fe(II) Complexes: Assessment of Electronic Structure Descriptions

**DOI:** 10.1021/acs.jctc.4c01331

**Published:** 2025-01-03

**Authors:** Mátyás Pápai

**Affiliations:** HUN-REN Wigner Research Centre for Physics, P.O. Box 49, H-1525 Budapest, Hungary

## Abstract

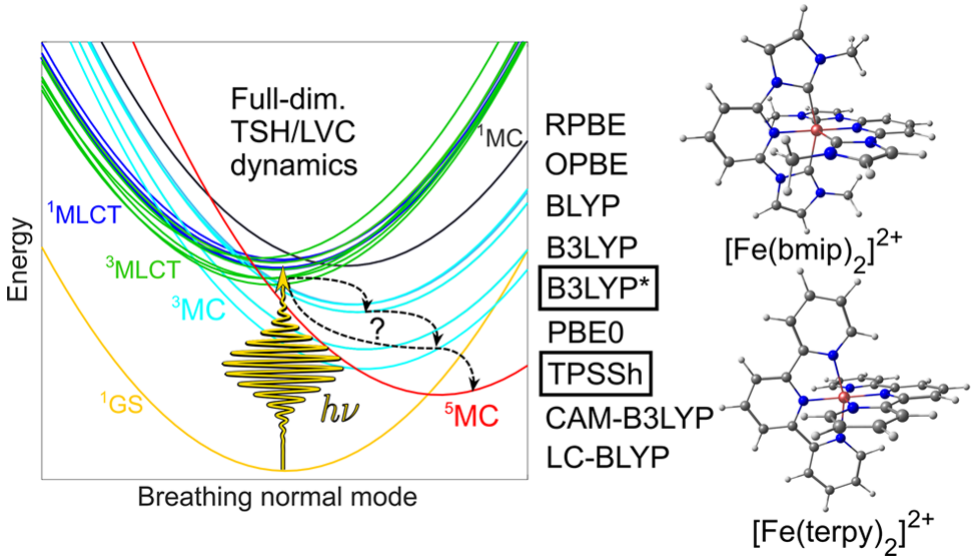

The assessment of electronic structure descriptions utilized in the simulation of the ultrafast excited-state dynamics of Fe(II) complexes is presented. Herein, we evaluate the performance of the RPBE, OPBE, BLYP, B3LYP, B3LYP*, PBE0, TPSSh, CAM-B3LYP, and LC-BLYP (time-dependent) density functional theory (DFT/TD-DFT) methods in full-dimensional trajectory surface hopping (TSH) simulations carried out on linear vibronic coupling (LVC) potentials. We exploit the existence of time-resolved X-ray emission spectroscopy (XES) data for the [Fe(bmip)_2_]^2+^ and [Fe(terpy)_2_]^2+^ prototypes for dynamics between metal-to-ligand charge-transfer (MLCT) and metal-centered (MC) states, which serve as a reference to benchmark the calculations (bmip = 2,6-bis(3-methyl-imidazole-1-ylidine)-pyridine, terpy = 2,2′:6′,2″-terpyridine). The results show that the simulated ultrafast population dynamics between MLCT and MC states with various spin multiplicities (singlet, triplet, and quintet) highly depend on the utilized DFT/TD-DFT method, with the percentage of exact (Hartree–Fock) exchange being the governing factor. Importantly, B3LYP* and TPSSh are the only DFT/TD-DFT methods with satisfactory performance, best reproducing the experimentally resolved dynamics for both complexes, signaling an optimal balance in the description of MLCT–MC energetics. This work demonstrates the power of combining TSH/LVC dynamics simulations with time-resolved experimental reference data to benchmark full-dimensional potential energy surfaces.

## 1 Introduction

Resolving ultrafast excited-state dynamics in molecules^1−4^ is an ultimate goal in modern physics and chemistry. In addition to its fundamental importance in natural sciences, such knowledge can also be exploited in various technological areas such as molecular data storage^5^ and the production of green energy based on solar energy conversion^6^ and photocatalysis.^7^ Photoactive transition-metal complexes^8^ are among the most-investigated molecules owing to their favorable properties, both in terms of applications (e.g., strong absorption in the visible region, long-lived excited states) and implementation of time-resolved X-ray scattering and spectroscopic experiments.^9^ Fe complexes are particularly appealing as they are cheap and environmentally friendly, and several of them possess advantageous excited-state profiles. Such properties are, e.g., the photoswitchable low-spin (LS) and high-spin (HS) states of Fe(II) polypyridines,^10,11^ making them promising candidates as molecular data storage devices^12,13^ and the long-lived metal-to-ligand charge-transfer (MLCT) states of Fe–N-heterocyclic carbenes,^14−18^ which could be potentially exploited in solar cells and photoctalysts.

Ultrafast excited-state dynamics are at the heart of photofunctional molecules, which determine their applicability and efficiency. Time-resolved (pump–probe) experiments utilizing ultrashort (∼ps–fs) pulses^19,20^ are powerful tools to resolve excited-state mechanisms; however, the analysis of the obtained data can be problematic, often leading to contradictory interpretations. Theory^21^—in particular, time-dependent approaches—can deliver valuable excited-state data that might be difficult to access by experimental techniques, due to, e.g., limited time resolution and/or overlapping signals. The interplay of experiments and theory thus has a crucial importance in deciphering all details of the ultrafast dynamics. Ideally, quantum dynamics (QD) would be the method of choice for simulating excited-state dynamics as it can deliver the exact solution by solving the time-dependent Schrödinger equation. However, for transition-metal complexes, QD simulations can only be performed in a drastically reduced dimension.^22−25^ Trajectory surface hopping (TSH), on the other hand, is operative in full dimension, as nuclear motion is treated classically, but quantum effects are inherently not taken into account (certain quantum phenomena can be introduced approximately such as electronic transitions and decoherence). Recently, we found for Fe complexes that full-dimensionality is by far more important than the rigorous treatment of quantum effects.^22,26−28^

**Figure 1:**
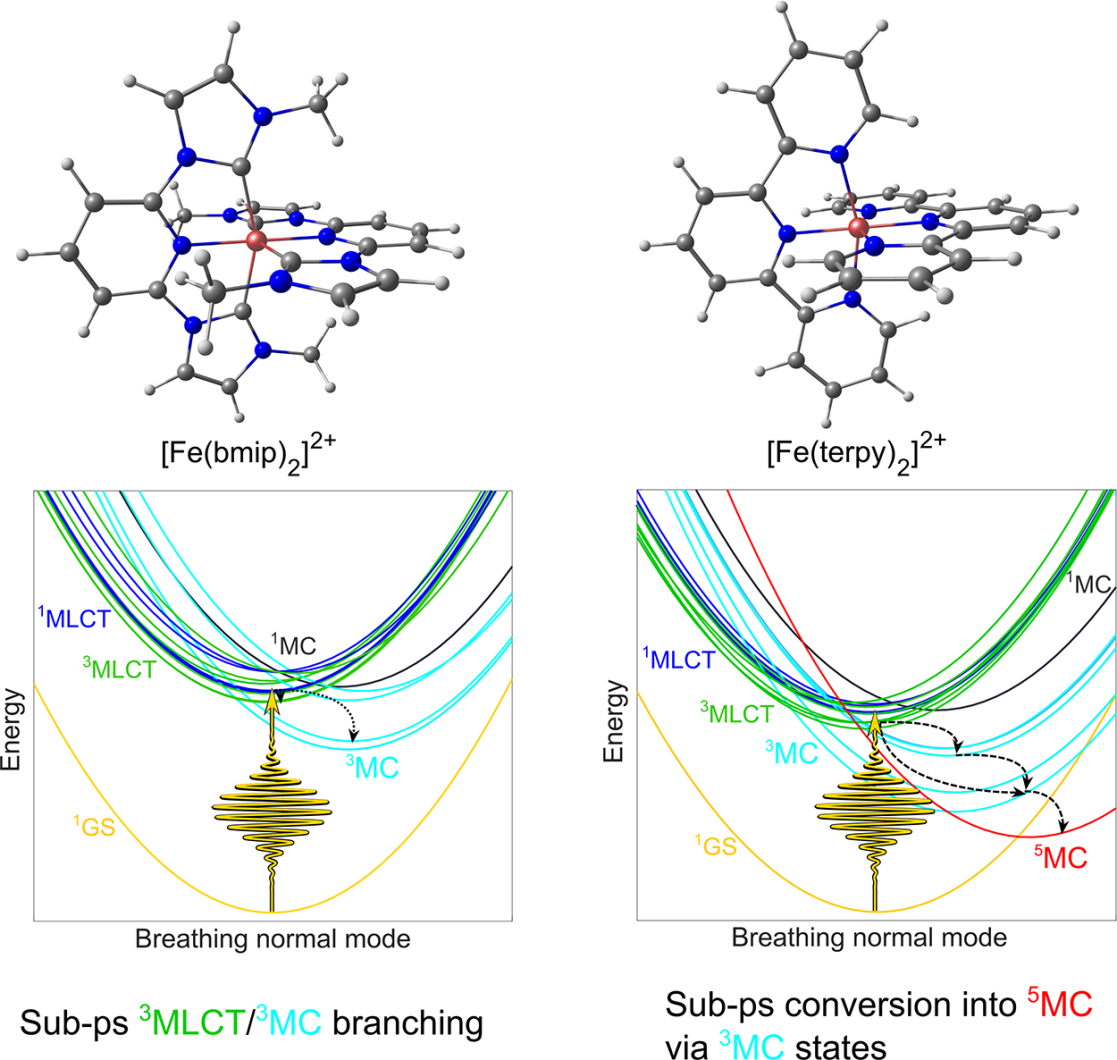
Molecular structure and schematics of the excited-state dynamics of the [Fe(bmip)_2_]^2+^ (left) and [Fe(terpy)_2_]^2+^ (right) complexes. The electronic excitation is considered to occur at the lowest optically active ^1^MLCT state. In both cases, the photophysics are dominated by MLCT–MC dynamics. In the case of [Fe(bmip)_2_]^2+^, the ^5^MC state is neglected, as it does not contribute to the dynamics.^29,30^ The data for the potential energy curves were taken from refs (26,28).

In our recent works,^26,28^ we simulated the excited-state dynamics of the [Fe(bmip)_2_]^2+^ and [Fe(terpy)_2_]^2+^ complexes using full-dimensional TSH (bmip = 2,6-bis(3-methyl-imidazole-1-ylidine)-pyridine, terpy = 2,2′:6′,2″-terpyridine). These two Fe(II) complexes are important prototypes for ultrafast dynamics between MLCT and metal-centered (MC) states triggered by MLCT excitation. The Fe-carbene [Fe(bmip)_2_]^2+^ complex is the first-discovered Fe-based photosensitizer^31^ with a ^3^MLCT lifetime of 9 ps, which is about 100 times larger than the one of Fe(II) polypyridines^32,33^ such as [Fe(terpy)_2_]^2+^ (∼100 fs), leading though to the population of the quintet HS MC state (^5^MC) on a subpicosecond time scale (see Figure 1). Moreover, for [Fe(bmip)_2_]^2+^, it turned out that the scenario is more complicated, with a fraction of the ^3^MLCT population converted into ^3^MC already on subpicosecond time scales.^30^ All of these mechanistic insights were obtained through time-resolved experiments. Importantly, our full-dimensional TSH simulations^26,28^ led to results in good agreement with the experiments and to improved mechanistic interpretations. However, from a methodological point of view, several aspects of the simulations governing the dynamics should be analyzed in greater detail. One of the most important factors impacting the simulated dynamics is the choice of electronic structure method to calculate the potential energy surfaces (PESs), on which the dynamics simulations are carried out. In this work, we exploit the existence of time-resolved experimental data to benchmark electronic structure (density functional) methods for the simulation of excited-stated dynamics of the [Fe(bmip)_2_]^2+^ and [Fe(terpy)_2_]^2+^ complexes.

## 2 Computational Methods

The utilized methodology is based on our previous works;^26,28^ we here briefly summarize the main points and focus on the details specific to the present work.

### 2.1 Electronic Structure Calculations

The PESs for the TSH dynamics are obtained by applying the linear vibronic coupling (LVC) model.^34,35^ The LVC potential is based on three approximations: (i) the harmonic oscillator approximation, (ii) the LVC approximation, meaning that the vibronic coupling elements depend linearly on normal mode coordinates, and (iii) the approximation of taking all excited-state vibrational frequencies to be equal to those corresponding to the ground state. The LVC parameters were determined by density functional theory (DFT) and time-dependent DFT (TD-DFT) calculations performed at the singlet ground-state (^1^GS) equilibrium geometry and geometries displaced by Δ*q*_*i*_ = ±0.5, with *q*_*i*_ being the dimensionless mass-frequency weighted normal mode coordinate for mode *i*; the gradients were computed analytically at the ^1^GS equilibrium geometry.

In our previous TSH/LVC works,^26,28^ we employed the hybrid B3LYP* exchange–correlation functional,^36^ which we selected based on our quantum chemical benchmarks^26,37^ and comparison to experimental reference data.^38,39^ We herein test the performance of several DFT/TD-DFT methods representing different rungs of Jacob’s ladder: GGAs–RPBE,^40,41^ OPBE,^40,42^ and BLYP,^43,44^ global hybrids–B3LYP^44−46^ and PBE0,^47^ meta-hybrid GGA−TPSSh, and range-separated hybrids—CAM-B3LYP^48^ and LC-BLYP;^49^ we included the double-hybrid B2PLYP functional as well; however, due to its high computational cost and the lack of implemented analytic TD-DFT gradients, we had to limit our assessment to the comparison of vertical excitation energies (which is presented in Section S1 and Tables S1–S6 in the Supporting Information). In all DFT/TD-DFT computations, we used the TZVP basis set.^50^ Solvation effects were not taken into account as they do not affect significantly the MLCT–MC energetics for the investigated Fe complexes.^26,28^ Two-electron integrals were approximated by using the resolution of identity approaches.^51,52^ In all TD-DFT calculations, we utilized the Tamm–Dancoff approximation (TDA).^53^ In Section S4 and Tables S7−S10 of the Supporting Information, we present an analysis of the effect of TDA on the calculated singlet–singlet and singlet–triplet TD-DFT excitation energies. We find that the effect is rather small, up to ca. 0.2 eV (but in most cases, significantly smaller), and importantly, it does not alter considerably the MLCT–MC energetics.

The spin–orbit couplings (SOCs) were calculated at the ^1^GS equilibrium geometry and were taken to be the same at any nuclear geometry. In the case of [Fe(bmip)_2_]^2+^, singlet and triplet states were included in the LVC models and the SOCs were calculated with the given DFT/TD-DFT method (at the ^1^GS geometry optimized by the same level of DFT). Accounting for scalar relativistic effects by utilization of the zeroth-order regular approximation (ZORA)^54,55^ did not lead to any significant differences in the calculated TD-DFT SOCs (see Section S5 in the Supporting Information). For [Fe(terpy)_2_]^2+^, however, the situation is more complicated as in addition the singlet and triplet states, quintets also have to be included. We employ our hybrid approach,^26,56^ according to which the LVC PESs are obtained by DFT/TD-DFT (using a restricted singlet reference for singlets and triplets but an unrestricted quintet reference for quintet states), but the SOCs are calculated by multiconfigurational second-order perturbation theory (CASPT2). The CASPT2 SOC matrix for [Fe(terpy)_2_]^2+^ was taken from ref (26) and was thus identical in all simulations (reindexing was though necessary as state ordering often differed for different TD-DFT methods). In the Supporting Information of ref (26), we validated the applied methodology both in terms of combining TD-DFT singlet–triplet and unrestricted DFT quintets (by comparing DFT/TD-DFT and CASPT2 PESs) and the correspondence of TD-DFT and CASPT2 states (which is an important requirement to combine DFT/TD-DFT PESs with CASPT2 SOCs). As in ref (26), similarity of the TD-DFT vs CASPT2 states allowed clear correspondence, with the exception of CAM-B3LYP, for which rather strong ^1^MLCT/^1^MC mixing was observed at the ground-state equilibrium geometry.

All DFT/TD-DFT calculations were carried out using the ORCA5.0 quantum chemistry software,^57,58^ with the exception of TPSSh TD-DFT analytic gradients, which were computed using ORCA6.0, as they became only available in this version.

### 2.2 TSH Dynamics Simulations

The full-dimensional TSH dynamics simulations were carried out in exactly the same way as in refs (26,28), only on different LVC potentials. We propagated the trajectories up to 1 ps using 0.5 and 0.005 fs time steps for the nuclear and electronic propagation, respectively. The TSH simulations were performed in the diagonal basis (which is obtained by full diagonalization of the diabatic LVC potential energy matrix, with the off-diagonal elements being the nuclear-coordinate-dependent nonadiabatic couplings and the nuclear-coordinate-independent SOCs). Initial conditions were sampled from a ground-state Wigner distribution and filtered using a stochastic algorithm based on a 0.1 eV wide energy window centered at the excitation energy to the lowest-lying optically active ^1^MLCT state (calculated at the ^1^GS equilibrium geometry) and taking into account the singlet oscillator strengths. This ^1^GS → ^1^MLCT excitation energy was also used to define the number of electronic states included in the LVC models and TSH simulations. For [Fe(bmip)_2_]^2+^, all states 1 eV above the lowest optically active ^1^MLCT state at the ^1^GS geometry were included in the calculations; the application of this criterion led to 14/16 singlets/triplets for RPBE, 12/15 for OPBE, 14/18 for BLYP, 16/18 for B3LYP, 14/21 for PBE0, 16/22 for CAM-B3LYP, and 14/21 for LC-BLYP. For [Fe(terpy)_2_]^2+^, the utilization of CASPT2 SOCs limited the number of states as further states would require expanding our active space, which would mean infeasibility for CASPT2 calculations. Nevertheless, we could still include all states ca. 0.5 eV above the lowest optically active ^1^MLCT, which should still be sufficient. For [Fe(terpy)_2_]^2+^, we included the lowest three quintet states for all DFT/TD-DFT methods and 13/18 singlets/triplets for RPBE, OPBE, and BLYP, 15/18 for B3LYP and PBE0, 13/18 for TPSSh, and 15/14 for LC-BLYP. For one of the singlet states of [Fe(terpy)_2_]^2+^, calculated by CAM-B3LYP at the ground-state equilibrium geometry, we observed rather strong ^1^MLCT/^1^MC mixing, which did not allow the assignment of DFT/TD-DFT vs CASPT2 states. Similarly, several of the lowest triplet states of [Fe(bmip)_2_]^2+^, calculated by TPSSh at the ground-state reference geometry, exhibited strong ^3^MLCT/^3^MC mixing, which would not allow the separation of the diabatic ^3^MLCT and ^3^MC populations. Therefore, in these two cases, we did not carry out TSH dynamics simulations. We note that for all other calculations, the MLCT or MC electronic character could be clearly identified. In the TSH simulations, we employed the decoherence correction scheme of Granucci et al.^59^ using a 0.1 au decoherence parameter value. We transformed the adiabatic (spin-diabatic) populations obtained from the TSH simulations to the diabatic basis of the given LVC model.^26,28^

All TSH dynamics simulations were carried out using the SHARC2.1 nonadiabatic molecular dynamics code.^60,61^

## 3 Results

The level of quantum chemistry is a crucial factor for all excited-state dynamics simulations.^62^ For transition-metal complexes, the state of the art of electronic structure theory is still heavily dominated by DFT/TD-DFT. While being computationally rather efficient, the PESs obtained by these methods may highly depend on the chosen exchange–correlation functional, in particular, when electronic states with different spin multiplicities are considered. Benchmarking DFT/TD-DFT results against higher-level calculations (e.g., CASPT2) and/or experimental data is thus valuable. However, such studies have been scarce for excited-state dynamics, which probe an extended region of PESs (in contrast to local equilibrium properties); this is especially true for transition-metal complexes, with only one sole effort so far known, focusing on optimally tuning range-separated functionals for an Fe complex.^63^ Herein, we assess the performance of functionals belonging to different rungs of Jacob’s ladder within the LVC framework making extensive use of the existence of high-quality time-resolved experimental data obtained by X-ray emission spectroscopy, which is a sensitive probe for electronic states of transition-metal complexes.^11,32,64^ We base our assessment on identifying qualitative features and time scales in the simulated and experimentally extracted population dynamics. We note that our approach can be considered as an interesting alternative to contrasting full-dimensional DFT/TD-DFT dynamics against those carried out at a higher level of quantum chemistry, such as CASPT2, which are currently not feasible for complexes such as [Fe(bmip)_2_]^2+^ and [Fe(terpy)_2_]^2+^. We mention, however, that this type of validation is aimed to complement conventional benchmarking against higher-level calculations, not to replace them.

### 3.1 [Fe(bmip)_2_]^2+^

**Figure 2:**
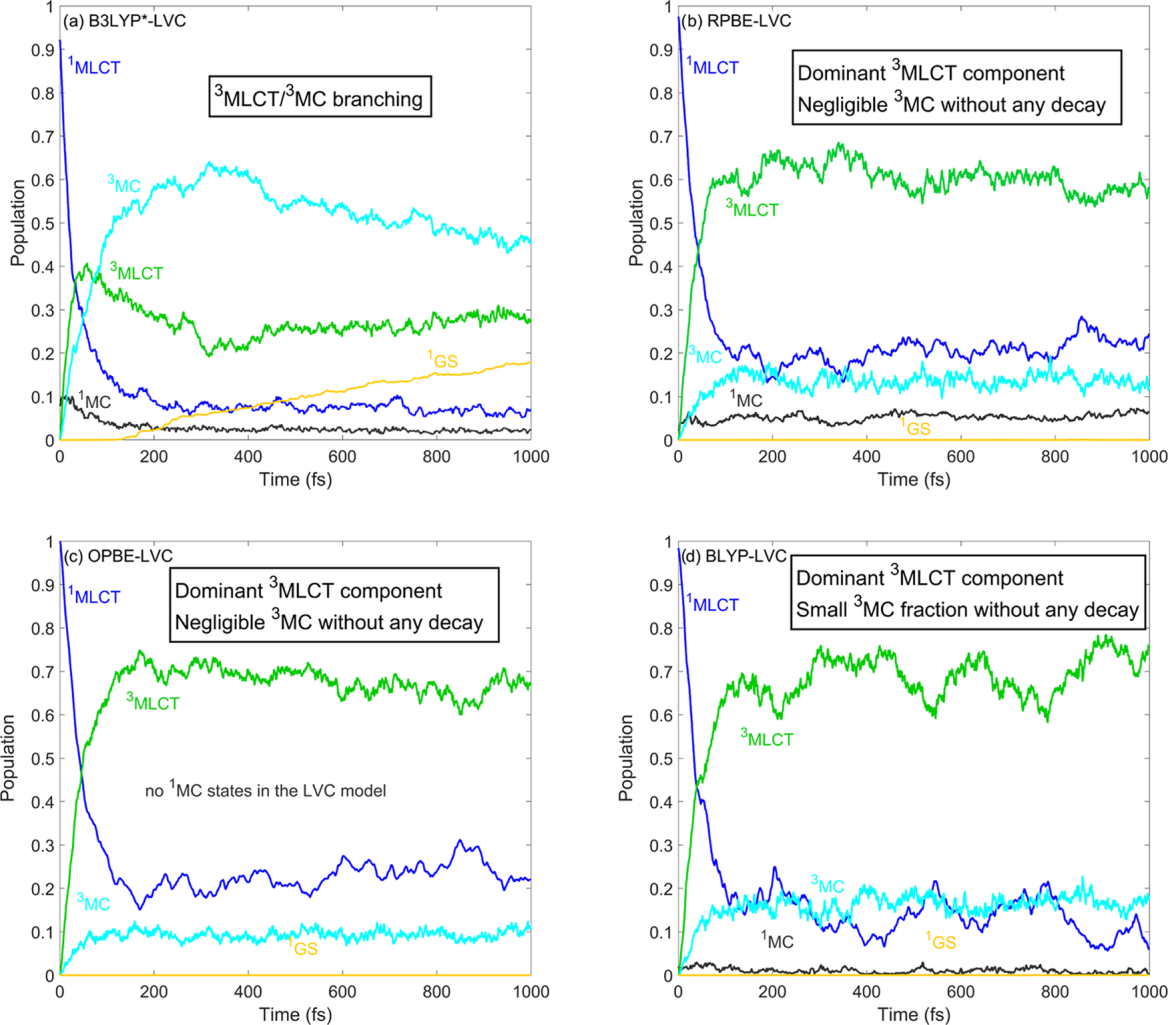
Simulated excited-state dynamics of [Fe(bmip)_2_]^2+^. The B3LYP* data shown in panel (a) were taken from ref (28). The shown populations were obtained by averaging over 267, 93, 135, and 78 trajectories for panels (a)–(d), respectively. In the case of OPBE, no ^1^MC states were included in the LVC model upon application of the energy criterion described in Section 2.2.

Figure 2 shows the excited-state dynamics of [Fe(bmip)_2_]^2+^, photoexcited to the lowest bright ^1^MLCT states, simulated by the RPBE, OPBE, and BLYP GGA functionals in comparison to our previous B3LYP* results (taken from ref (28)). As is clear from the figure, all three GGA-LVC methods yield rather similar results but different from those obtained by B3LYP*-LVC. Although we observe a ^3^MLCT/^3^MC branching, it becomes rather weak as the ^3^MC component becomes almost negligible, and no ^3^MC decay and ^1^GS population growth are seen; furthermore, a small but non-negligible fraction of excited-state population is stuck in the ^1^MLCT states. Overall, the B3LYP* results are more consistent with the time-resolved experiment, taken as the reference, which is clearly indicative of a ^3^MLCT/^3^MC branching and ^3^MC decay, already on subpicosecond time scales.^30^

**Figure 3:**
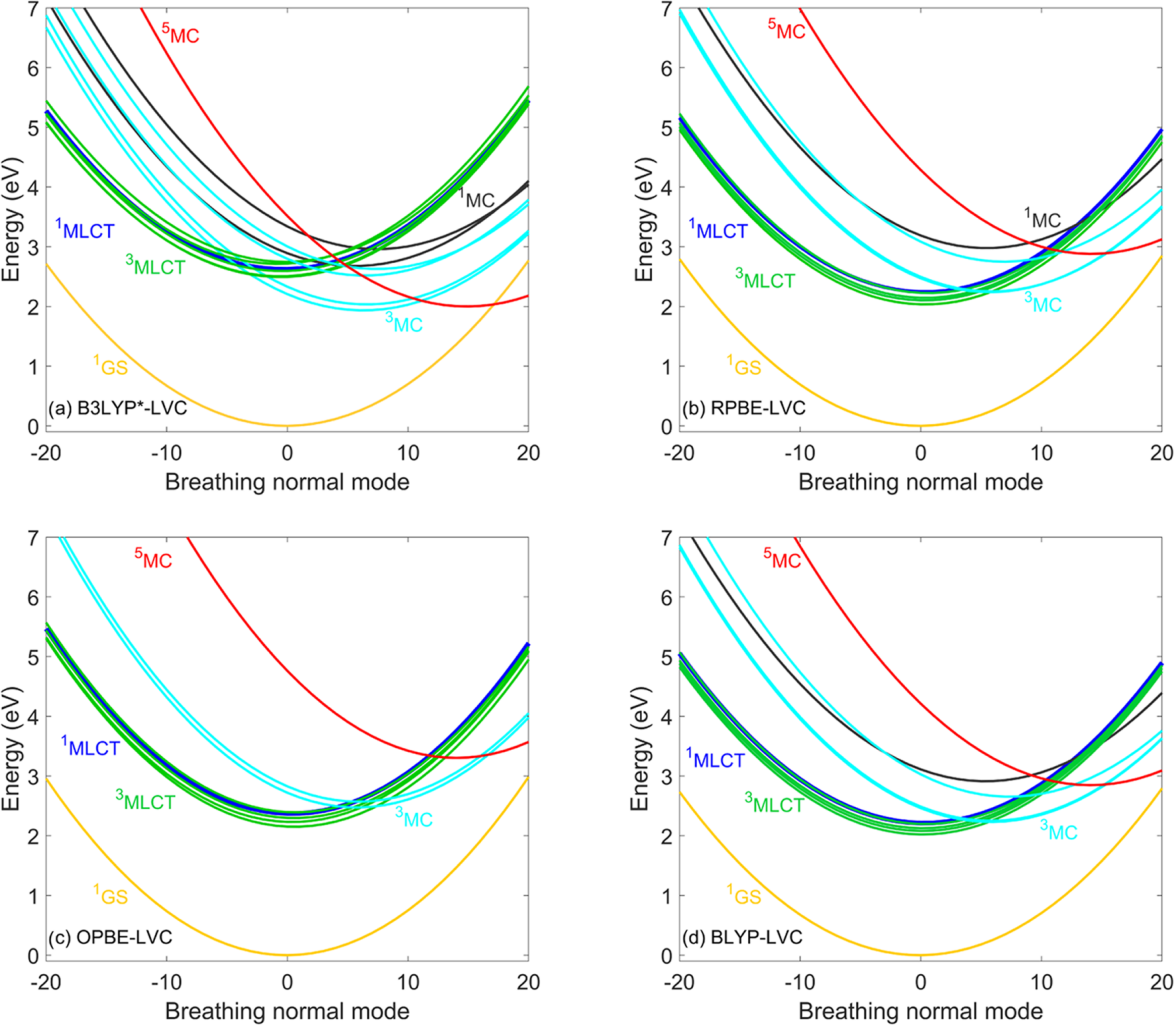
Diabatic LVC potentials of [Fe(bmip)_2_]^2+^ calculated by the (a) B3LYP*, (b) RPBE, (c) OPBE, and (d) BLYP exchange–correlation functionals. For improved visibility, only the lowest-lying MLCT states are shown. Nuclear displacements are given in dimensionless mass-frequency weighted normal mode coordinates.

We now analyze the results of our dynamics simulations in terms of the LVC potentials along the Fe-ligand breathing mode (which is known to be the dominant mode for MLCT–MC dynamics^22,27,33,65^) shown in Figure 3. We find that the energetic separation of ^3^MLCT–^3^MC potentials practically vanishes in the GGA cases, while for B3LYP*, the separation of the minima is larger than 0.5 eV, with the ^3^MC being lower. We identify this modulation of ^3^MLCT–^3^MC energetics as the main factor responsible for the drastic decrease in the weight of the ^3^MC component, observed for the GGA methods (see Figure 2b–d). We attribute the energy modulation to the inclusion of the exact (Hartree–Fock) exchange in the hybrid B3LYP*, which affects both MLCT and MC states but with opposite signs; therefore, the separation of these states changes significantly. The quintet ^5^MC state was experimentally found not to contribute to the excited-state dynamics of [Fe(bmip)_2_]^2+^; we have thus not included it in the simulations. Nevertheless, we display its LVC PES in Figure 3 (red curves) in order to assess qualitatively its potential impact on the dynamics. For the three GGA methods (Figure 3b–d), the ^5^MC potential is significantly displaced from the region at which the dynamics predominantly occur, i.e., around the ^3^MLCT/^3^MC minima and the ^3^MLCT/^3^MC crossings; therefore, the ^5^MC states can here be safely neglected. In the case of B3LYP* (Figure 3a), the ^5^MC states are still not expected to affect the ^3^MLCT/^3^MC branching; however, they might slightly alter the ^3^MC decay, as the crossing with the lower ^3^MC PESs becomes more accessible than for the GGAs. Still, the B3LYP* excited-state description is the one that is more consistent with the time-resolved experiments, as it reproduces the ^3^MLCT/^3^MC branching, which is the most striking feature of the dynamics of [Fe(bmip)_2_]^2+^.

**Figure 4:**
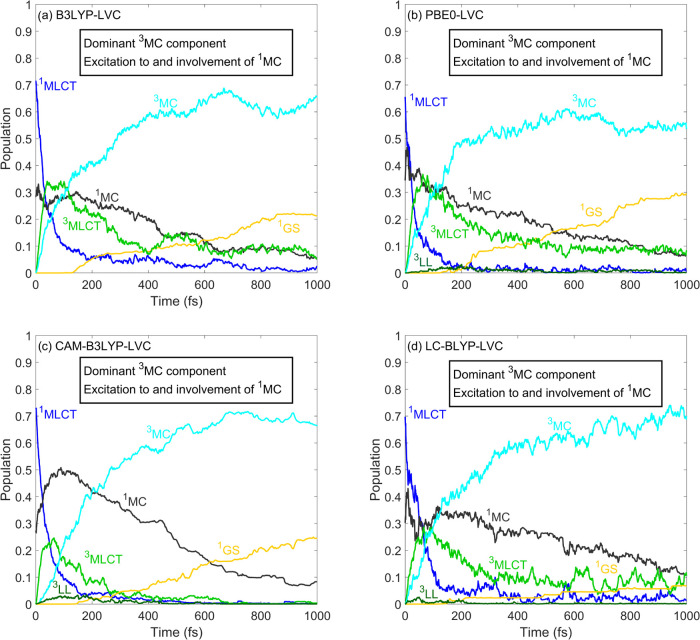
Simulated excited-state dynamics of [Fe(bmip)_2_]^2+^. The shown populations were obtained by averaging over 143, 87, 127, and 89 trajectories for panels (a)–(d), respectively.

We now present and discuss our results obtained by the B3LYP and PBE0 global hybrid functionals and the CAM-B3LYP and LC-BLYP range-separated hybrids. As seen in Figure 4, in all cases, a non-negligible initial ^1^MC population appears, with the ^1^MLCT still being the major component of the excited-state manifold generated by the electronic excitation. However, the ^1^MC states here do play a role in the relaxation mechanism, which was not the case for the B3LYP* functional and the GGA methods (see Figure 2). Furthermore, the ^3^MLCT/^3^MC branching disappears, as the ^3^MLCT population decays rapidly; only up to ca. 5% remains at the final 1 ps step of the simulations. The ^3^MC component, on the other hand, becomes dominant, and no population decay is observed. These results are in clear disagreement with the experimental observations. We note that for PBE0, CAM-B3LYP, and LC-BLYP, ^3^LL states (corresponding to ligand–ligand transitions) became included in the LVC models; thus, ^3^LL population curves appear in Figure 4b–d, albeit these populations are so small that they are negligible (this is because the ^3^LL states are located in higher-energy regions that are energetically not accessible for the dynamics).

**Figure 5:**
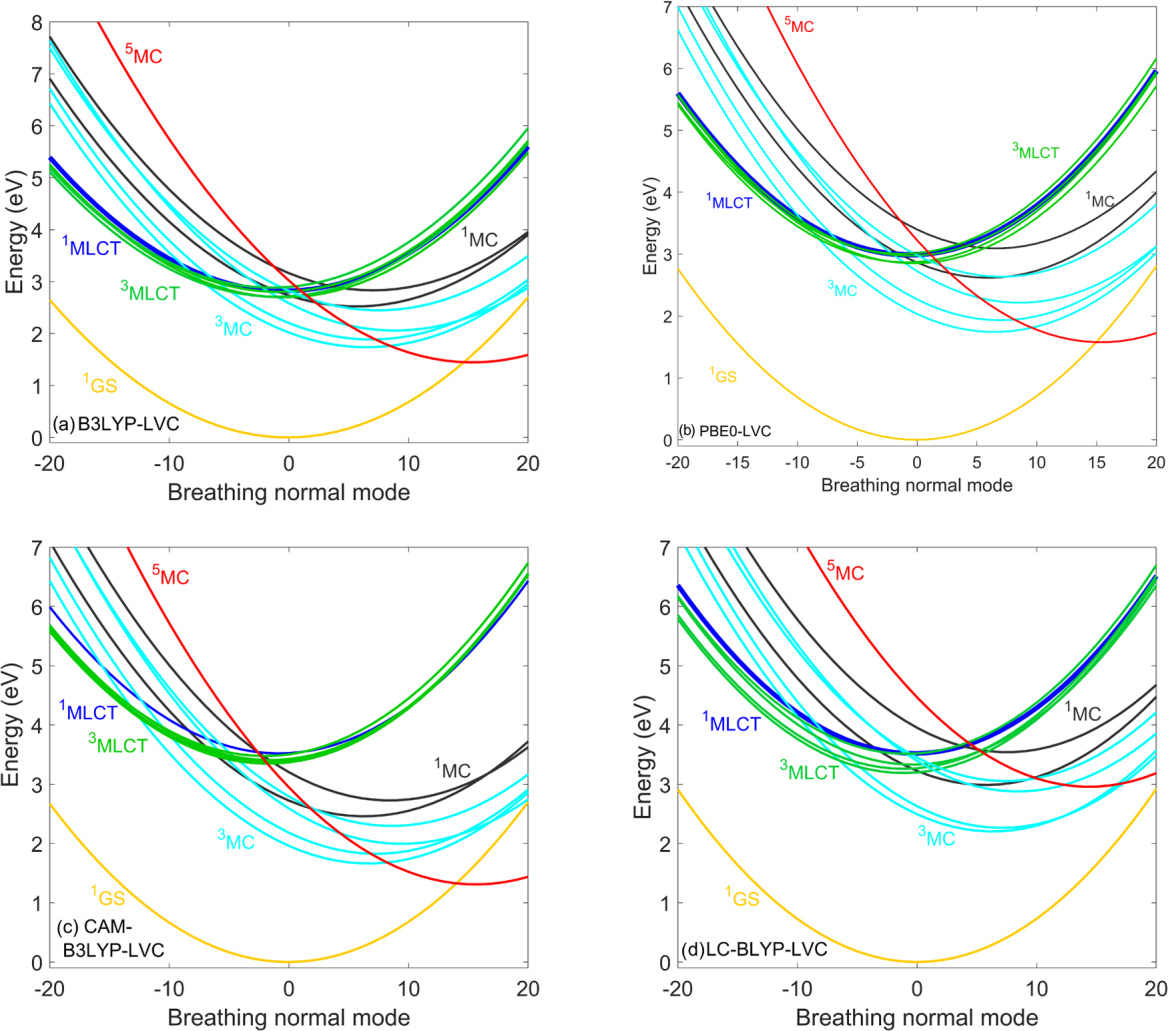
Diabatic LVC potentials of [Fe(bmip)_2_]^2+^ calculated by the (a) B3LYP, (b), PBE0, (c) CAM-B3LYP, and (d) LC-BLYP exchange–correlation functionals For improved visibility, only the lowest-lying MLCT states are shown. Nuclear displacements are given in dimensionless mass-frequency weighted normal mode coordinates.

Figure 5 shows the LVC potentials of [Fe(bmip)_2_]^2+^ obtained by the global and range-separated hybrid DFT/TD-DFT methods along the breathing normal mode. In general, the MC states are lowered with respect to the MLCTs compared to the B3LYP* potentials shown in Figure 3a. This is the reason for the two main features observed in the simulated dynamics, namely, the involvement of ^1^MC as they get below the bright ^1^MLCT at the ground-state equilibrium geometry and the short ^3^MLCT lifetime. In addition, for B3LYP, PBE0, and CAM-B3LYP, the potential role of the ^5^MC state increases as its PES is lowered and thus moves into the region, which is mostly accessed during the dynamics; this can be illustrated by the location of the crossing between the ^5^MC potential with those of the lowest ^3^MLCT and ^3^MC states, which is very close to the ^3^MLCT and ^3^MC minima. For LC-BLYP, however, the ^5^MC curve is placed significantly higher; the ^5^MC minimum actually is located higher than the minimum of the lowest ^3^MC state, similarly to the GGA cases (see Figure 3b–d), for which the ^5^MC state can safely be neglected. We attribute the drastic difference between the ^5^MC curves calculated by the two range-separated hybrids CAM-B3LYP and LC-BLYP shown in Figure 5c,d to the fixed fraction of the exact exchange that is 19% for CAM-B3LYP (very close to B3LYP with 20% exact exchange) but 0% for LC-BLYP. We therefore arrive at the conclusion regarding the ^5^MC state that it would have a major effect on the dynamics for B3LYP, PBE, and CAM-B3LYP, but not for LC-BLYP.

As mentioned in Section 2.2, we could not directly assess the performance of the meta-hybrid GGA functional TPSSh for the dynamics of [Fe(bmip)_2_]^2+^, as several of the calculated diabatic triplet states had a mixed ^3^MLCT/^3^MC character. However, this observation implies that ^3^MLCT/^3^MC branching would occur in the TPSSh dynamics, as the dynamics would eventually lead to the population of the lowest states that possess mixed ^3^MLCT/^3^MC character. In Figure S1 of the Supporting Information, we compare the adiabatic B3LYP* and TPSSh potential energy surfaces of [Fe(bmip)_2_]^2+^ along the breathing mode. We find rather good agreement, and interestingly, the energy difference between the ^3^MLCT and ^3^MC minima calculated by TPSSh is reduced (the ^3^MC minimum is still the lower-lying but only by ∼0.2–0.3 eV as opposed to the ∼0.5–0.6 eV energy difference for B3LYP*). This suggests that the ^3^MLCT/^3^MC branching ratio would move in favor of ^3^MLCT in better agreement with the experiment. Furthermore, due to the smaller fraction of the exact exchange in TPSSh as compared to that of B3LYP* (10% vs 15%), the lowest ^5^MC state is shifted up by ∼0.5 eV (see Table S3 in the Supporting Information), meaning that the involvement of the quintets for TPSSh can surely be ruled out. We note, however, that in contrast to the B3LYP* results, the considerations presented here do not come from explicit TPSSh dynamics simulations and that the B3LYP* dynamics are in rather good qualitative agreement with those extracted experimentally.

### 3.2 [Fe(terpy)_2_]^2+^

[Fe(terpy)_2_]^2+^ is a member of the family of Fe(II) polypyridines, which are known to undergo a light-induced spin transition from the singlet GS to the metastable quintet ^5^MC state within a few hundred femtoseconds.^32,33,66,67^ However, the mechanism of the photoswitching as proposed by time-resolved experiments has been controversial and thus has been debated over a decade whether the ^5^MC state is populated directly from the ^3^MLCT manifold or sequentially, i.e., ^3^MLCT → ^3^MC → ^5^MC. Recently, we clarified the excited-state mechanism of Fe(II) polypyridines by simulating the excited-state dynamics of [Fe(terpy)_2_]^2+^ using full-dimensional TSH simulations carried out on PESs calculated by B3LYP*-LVC.^26^ We note that shortly afterward, two papers on the [Fe(bipy)_3_]^2+^ complex were published (bipy = 2,2′-bipyridine), reporting ultrafast dynamics simulated by full-dimensional TSH^68^ and reduced-dimensional quantum dynamics.^27^ Importantly, all three dynamics studies consistently identify the sequential mechanism as the dominant one.

**Figure 6:**
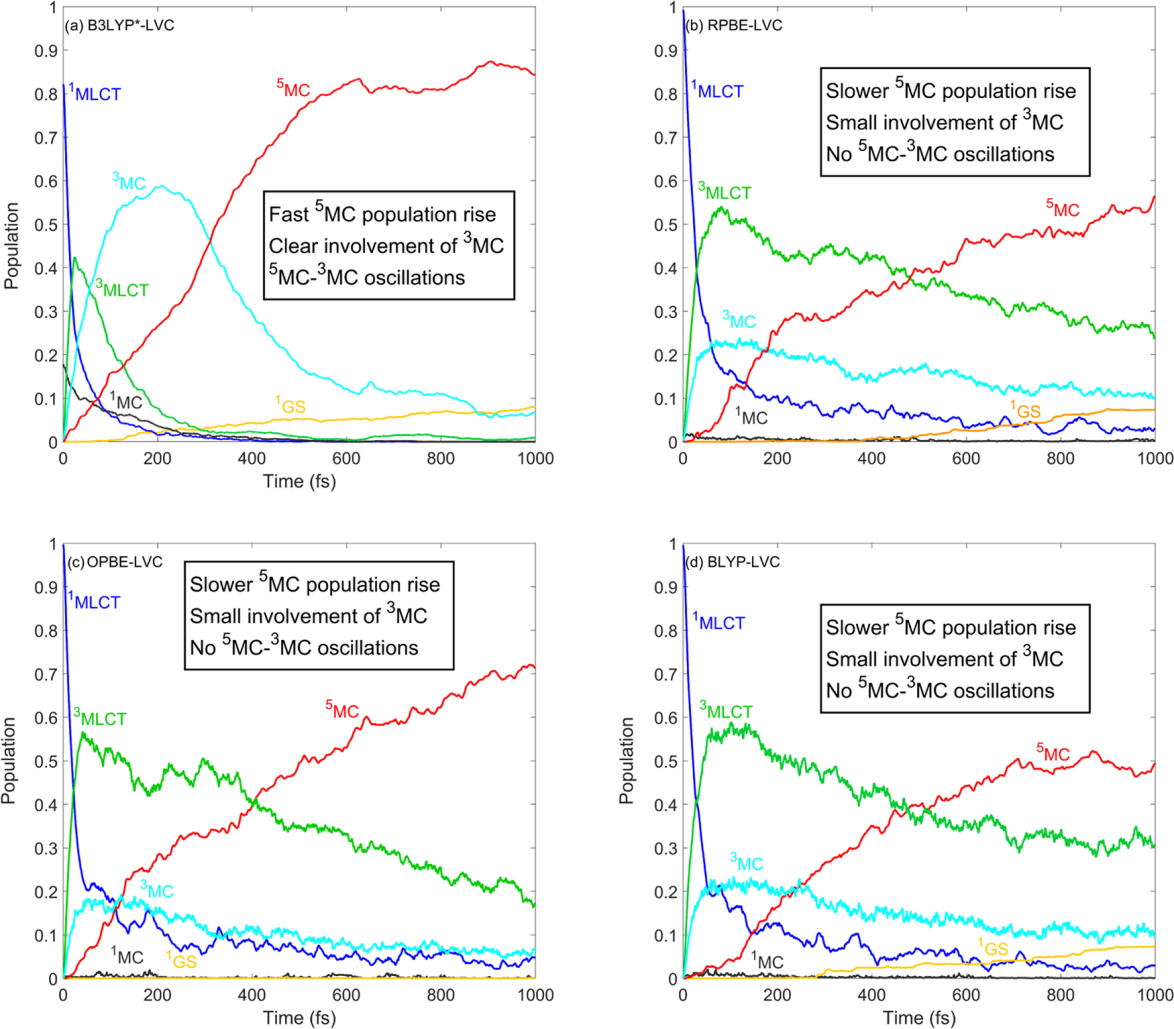
Simulated excited-state dynamics of [Fe(terpy)_2_]^2+^. The B3LYP* data shown in panel (a) were taken from ref (26). The shown populations were obtained by averaging over 716, 164, 125, and 124 trajectories for panels (a)–(d), respectively.

**Figure 7:**
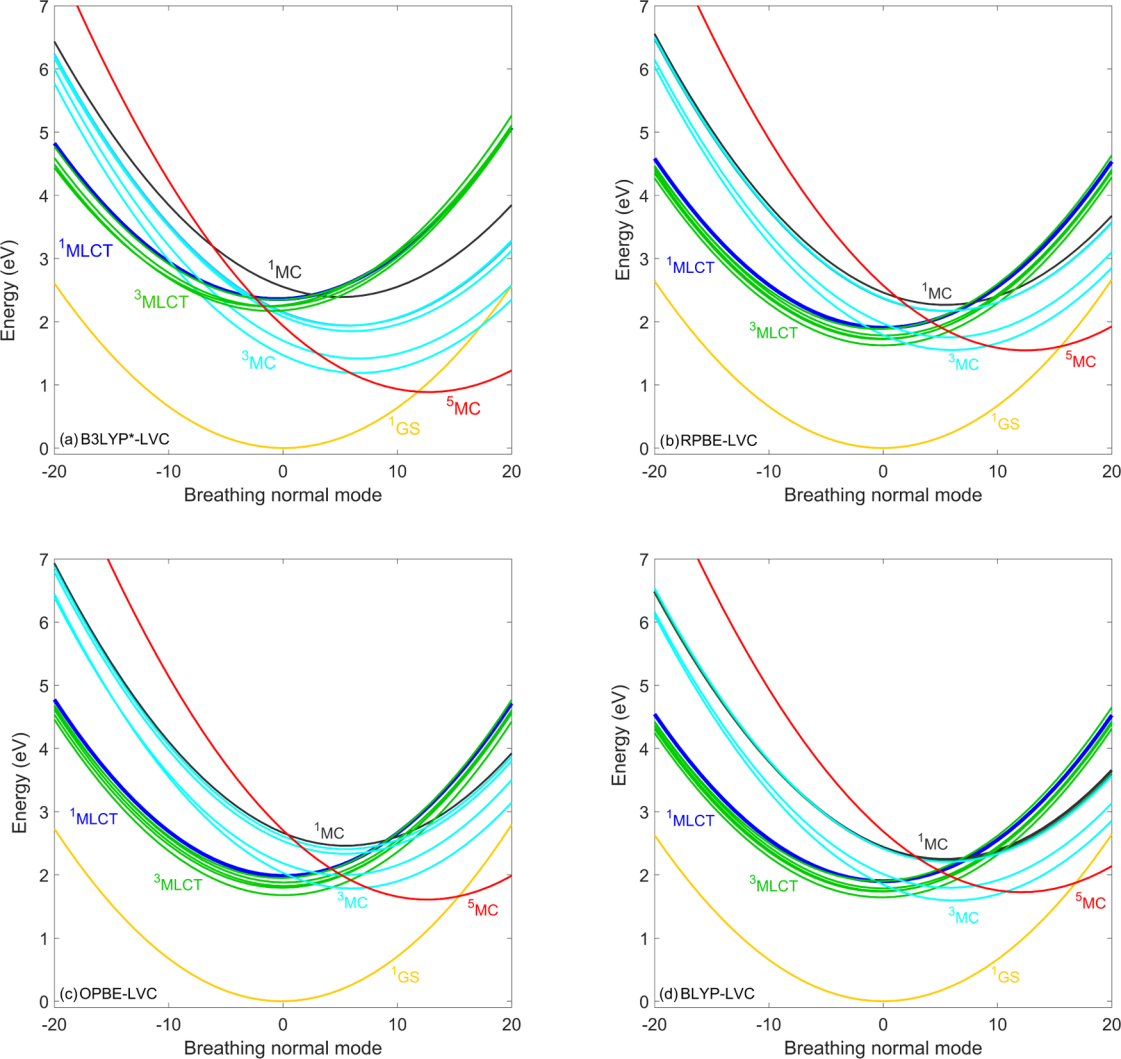
Diabatic LVC potentials of [Fe(terpy)_2_]^2+^ calculated by the (a) B3LYP*, (b) RPBE, (c) OPBE, and (d) BLYP exchange–correlation functionals. For improved visibility, only the lowest-lying MLCT states are shown. Nuclear displacements are given in dimensionless mass-frequency weighted normal mode coordinates.

We now assess how our simulated dynamics for [Fe(terpy)_2_]^2+^ obtained by the other DFT/TD-DFT methods compare with our previous B3LYP*-LVC results, which were found to agree with the most extensively analyzed time-resolved experimental data.^33^ We note that in contrast to the case of [Fe(bmip)_2_]^2+^, for [Fe(terpy)_2_]^2+^, quintet states cannot be neglected, as they clearly play an active role in the photorelaxation mechanism. Figure 6 shows the excited-state dynamics of [Fe(terpy)_2_]^2+^ simulated by B3LYP* and the three GGA methods, RPBE, OPBE, and BLYP. As seen in Figure 6, all three GGA methods again yield rather similar population dynamics but very different from the B3LYP* results: the ^3^MLCT manifold is longer lived, the ^5^MC population rise is slower, and the ^3^MC intermediate states do not play any dominant role in the photorelaxation dynamics. These differences are well explained by the PESs shown in Figure 7: for GGAs, the minima of the ^3^MLCT/^3^MC/^5^MC potentials are located at rather similar energies, while for B3LYP*, there is a significant lowering in the direction of ^3^MLCT → ^3^MC → ^5^MC (in line with the sequential mechanism). Importantly, this also has the consequence that the crossing of the ^3^MLCT/^3^MC and ^3^MLCT/^5^MC potentials is displaced from the ^3^MLCT minimum geometry, which explains the slower dynamics of ^3^MLCT and ^5^MC states and the significantly decreased ^3^MC population (in comparison to the B3LYP* case, see Figure 6).

**Figure 8:**
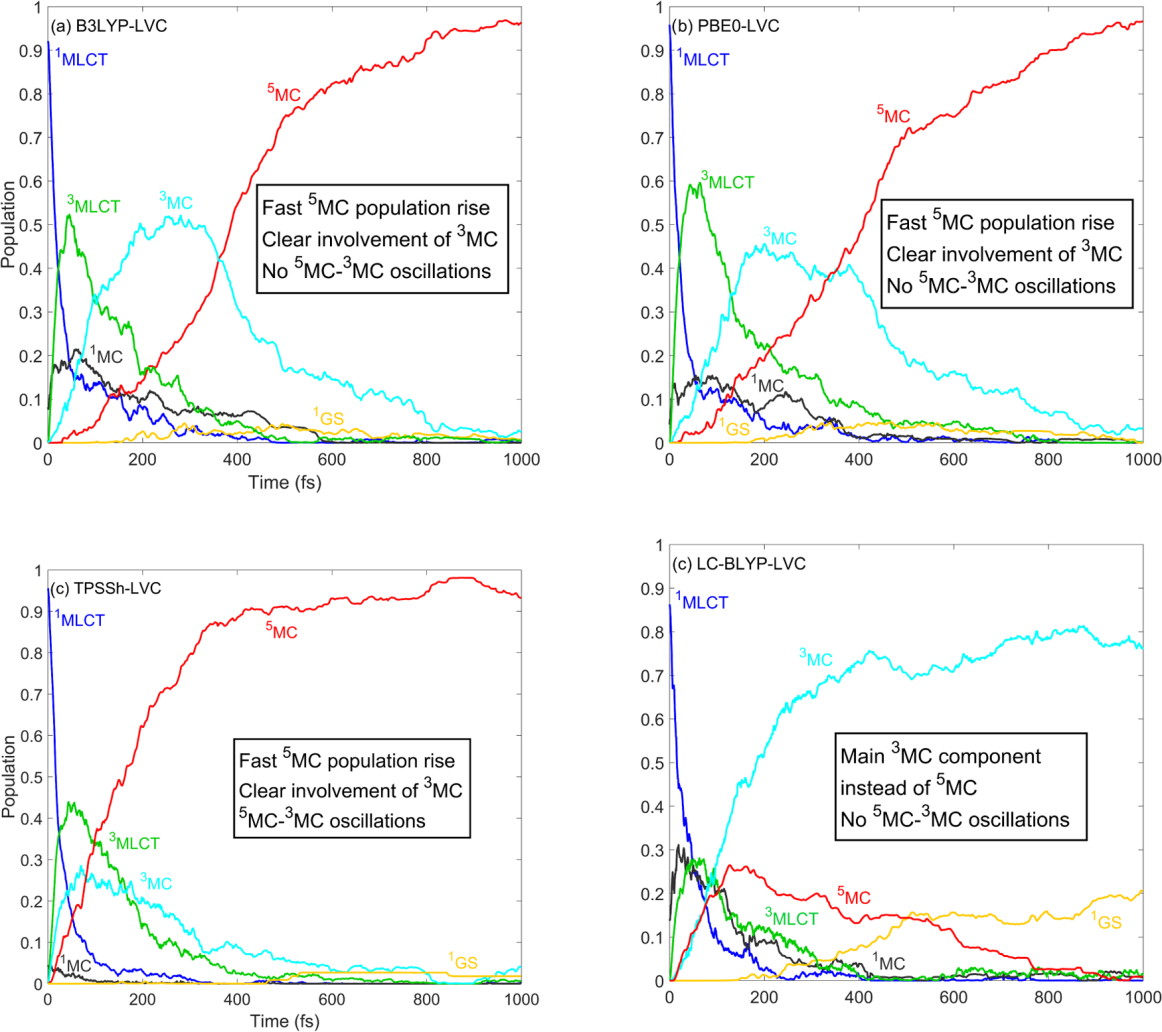
Simulated excited-state dynamics of [Fe(terpy)_2_]^2+^. The shown populations were obtained by averaging over 108, 110, 111, and 117 trajectories for panels (a)–(d), respectively.

**Figure 9:**
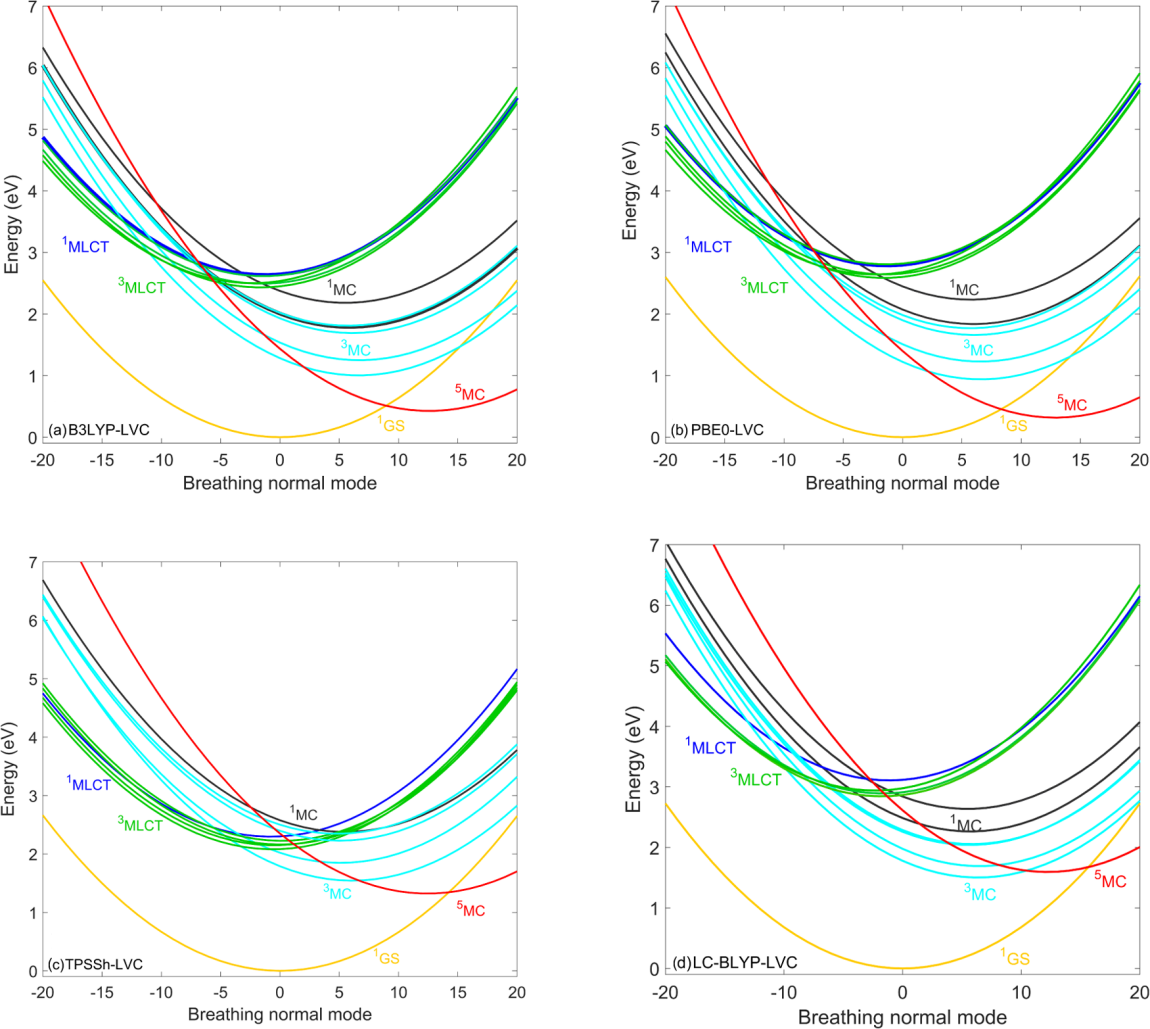
Diabatic LVC potentials of [Fe(terpy)_2_]^2+^ calculated by the (a) B3LYP, (b) PBE0, (c) TPSSh, and (d) LC-BLYP exchange–correlation functionals. For improved visibility, only the lowest-lying MLCT states are shown. Nuclear displacements are given in dimensionless mass-frequency weighted normal mode coordinates.

Finally, we analyze and discuss the results obtained for [Fe(terpy)_2_]^2+^ obtained by the global, meta-GGA, and range-separated hybrid functionals. Figure 8 shows the population dynamics of [Fe(terpy)_2_]^2+^ simulated by B3LYP, PBE0, TPSSh, and LC-BLYP. We here recall that, as noted in the Computational Methods section, we did not carry out TSH dynamics simulations on [Fe(terpy)_2_]^2+^ using CAM-B3LYP-LVC PESs due to the observed ^1^MLCT/^1^MC mixing at the ground-state geometry (which did not allow matching of the DFT/TD-DFT and CASPT2 states). The population dynamics of [Fe(terpy)_2_]^2+^ simulated by the two global hybrids, B3LYP and PBE0, shown in Figure 8a,8b, are in good overall agreement with the B3LYP* results,^26^ as well as the time-resolved XES experiment.^33^ Namely, all of these data are consistent with a ^1, 3^MLCT → ^3^MC → ^5^MC photorelaxation mechanism and the simulated time scales agree for B3LYP, B3LYP*, and PBE0. Furthermore, the nonexponential character of the ^5^MC population rise is also reproduced by B3LYP and PBE0. However, the ^5^MC–^3^MC oscillations caused by coherent vibrational motion along the Fe–N breathing mode in the ^5^MC states^26,33,67^ do not appear in our B3LYP and PBE0 results. One possible reason for the absence of the oscillations is the loss of vibrational coherence, i.e., the ^5^MC population rise occurs on a slower time scale as compared to the vibrational period of ∼300 fs; however, this is clearly not the case as seen in Figure 8a,b. Another more likely explanation in the present case is that the trajectories while propagating in the ^5^MC manifold do not reach the ^5^MC/^3^MC intersection, at which population transfer can occur. Analysis of the B3LYP and PBE0 PESs shown in Figure 9a,b and comparison to the B3LYP* ones (Figure 7a) support this interpretation; as for B3LYP and PBE0, the ^5^MC/^3^MC crossing gets significantly displaced from the ^5^MC minimum. The TPSSh result shown in Figure 8c also reproduces the time scales (in fact, the ^5^MC population rise is even faster than the B3LYP*-simulated one, closer to the experimentally resolved rate) and the ^5^MC–^3^MC oscillations; however, no clear exponential character can be identified in the TPSSh ^5^MC population curve. Finally, we discuss the population dynamics of [Fe(terpy)_2_]^2+^ simulated by the range-separated LC-BLYP method. As seen in Figure 8c, these results for [Fe(terpy)_2_]^2+^ are very different (even qualitatively) from those obtained by all other methods, as the ^5^MC population decays on a subpicosecond time scale and ^3^MC emerges as the dominant excited-state manifold at 1 ps. This is clearly in contradiction with the experimental data, evidencing that all excited-state population is converted into ^5^MC on a subpicosecond time scale; specifically, the ^3^MC population is depleted in ∼500 fs. We attribute the vanishing ^5^MC population in the LC-BLYP simulation to the significantly increased energy of the ^5^MC states relative to those of the ^3^MCs. As seen in Figure 9, the ^5^MC potentials calculated by LC-BLYP are shifted by 1.5 eV compared to the ^5^MC PESs calculated by B3LYP and PBE0, which places them above the ^3^MC states; this explains the strikingly different population dynamics obtained by the LC-BLYP and B3LYP/PBE0 simulations.

## 4 Discussion and Conclusions

The presented full-dimensional LVC-TSH simulations for both [Fe(bmip)_2_]^2+^ and [Fe(terpy)_2_]^2+^ demonstrate a high degree of dependence of the population dynamics on the utilized electronic structure (DFT/TD-DFT) method. We note that the observed differences stem dominantly from vertical shifts of the excited-state PESs (controlled in the LVC model by the excitation energies calculated at the ground-state equilibrium geometry), where the effect is by far more significant than differences seen in the interstate couplings. Importantly, B3LYP* and TPSSh are the only functionals that reproduce the most important aspects of the experimentally observed dynamics, indicating an accurate description of MLCT–MC energetics. Our assessment reveals that the exact (Hartree–Fock) exchange is the decisive factor determining the simulated dynamics. Pure GGA methods (RPBE, OPBE, BLYP) that do not contain any exact exchange tend to overstabilize MLCT states, which led to failures such as lack of any dominant subpicosecond ^3^MC component for [Fe(bmip)_2_]^2+^ and thus hardly any observable ^3^MLCT/^3^MC branching. For [Fe(terpy)_2_]^2+^, the ^5^MC population rise obtained by GGAs is too slow and importantly, the population dynamics are rather exponential with the absence of any ^5^MC–^3^MC oscillations, which contradicts both the time-resolved experimental XES data and our B3LYP* results. In fact, the obtained dynamics shown in Figure 6b–d are reminiscent of our B3LYP* results when the ^3^MC states were excluded in the simulation;^26^ this shows that the ^3^MC states do not play an active role in the excited-state mechanism as constructed from the GGA simulations, and the ^5^MC is populated directly from the ^3^MLCTs, which is in disagreement with all recent theoretical works^26,27,68^ on Fe(II) polypyridines and the time-resolved XES data.^32,33^

It is thus clear that a more balanced description of the MLCT–MC energetics is required in order to accurately simulate the excited-state dynamics. A straightforward approach is to utilize hybrid exchange–correlation functionals that include a certain amount of exact exchange. However, we found that the exact exchange within the tested B3LYP, PBE0, and CAM-B3LYP functionals is too high and that the MC states are those that are now overstabilized. Specifically, for [Fe(bmip)_2_]^2+^, this is reflected in the rapidly decaying ^1,3^MLCT population; thus, the experimentally observed ^3^MLCT/^3^MC branching is again not reproduced. In addition, we found based on the potentials along the principal breathing mode of [Fe(bmip)_2_]^2+^ calculated by the hybrid functionals B3LYP, PBE0, and CAM-B3LYP that the ^5^MC PES is incorrectly located in a region where it could in fact play an active role in the dynamics (while it is known experimentally^30^ that the ^5^MC state is not involved in the photorelaxation of [Fe(bmip)_2_]^2+^). For [Fe(terpy)_2_]^2+^, the excited-state description by the B3LYP and PBE0 hybrids is significantly better (as assessed by comparison to the XES experiment) but still not completely convincing as the ^5^MC–^3^MC oscillations present in both the experimental XES data^33^ and our B3LYP* dynamics^26^ are not observed. In contrast, B3LYP* correctly reproduces all important aspects of the dynamics for both [Fe(bmip)_2_]^2+^ and [Fe(terpy)_2_]^2+^, signaling that an optimal balanced description of MLCT–MC energetics is achieved. Regarding some aspects, TPSSh performs even better than B3LYP*, i.e., the relative ^3^MLCT–^3^MC energetics controlling the branching ratio for [Fe(bmip)_2_]^2+^ and the time scale of the ^5^MC population rise for [Fe(terpy)_2_]^2+^; on the other hand, we could not directly assess the TPSSh dynamics for [Fe(bmip)_2_]^2+^, and the nonexponential character of the ^5^MC population curve for [Fe(terpy)_2_]^2+^ is not reproduced. Therefore, based on the data obtained in the present work, we identify B3LYP* and TPSSh as the most reliable DFT/TD-DFT methods for the simulation of excited-state dynamics of [Fe(bmip)_2_]^2+^ and [Fe(terpy)_2_]^2+^. Based on the comparison of B2PLYP vs B3LYP*/TPSSh vertical excitation energies presented in the Supporting Information (see Section S1 and Tables S1−S6), we identify the performance of the B2PLYP DFT/TD-DFT method to be inferior to those of B3LYP* and TPSSh.

**Figure 10:**
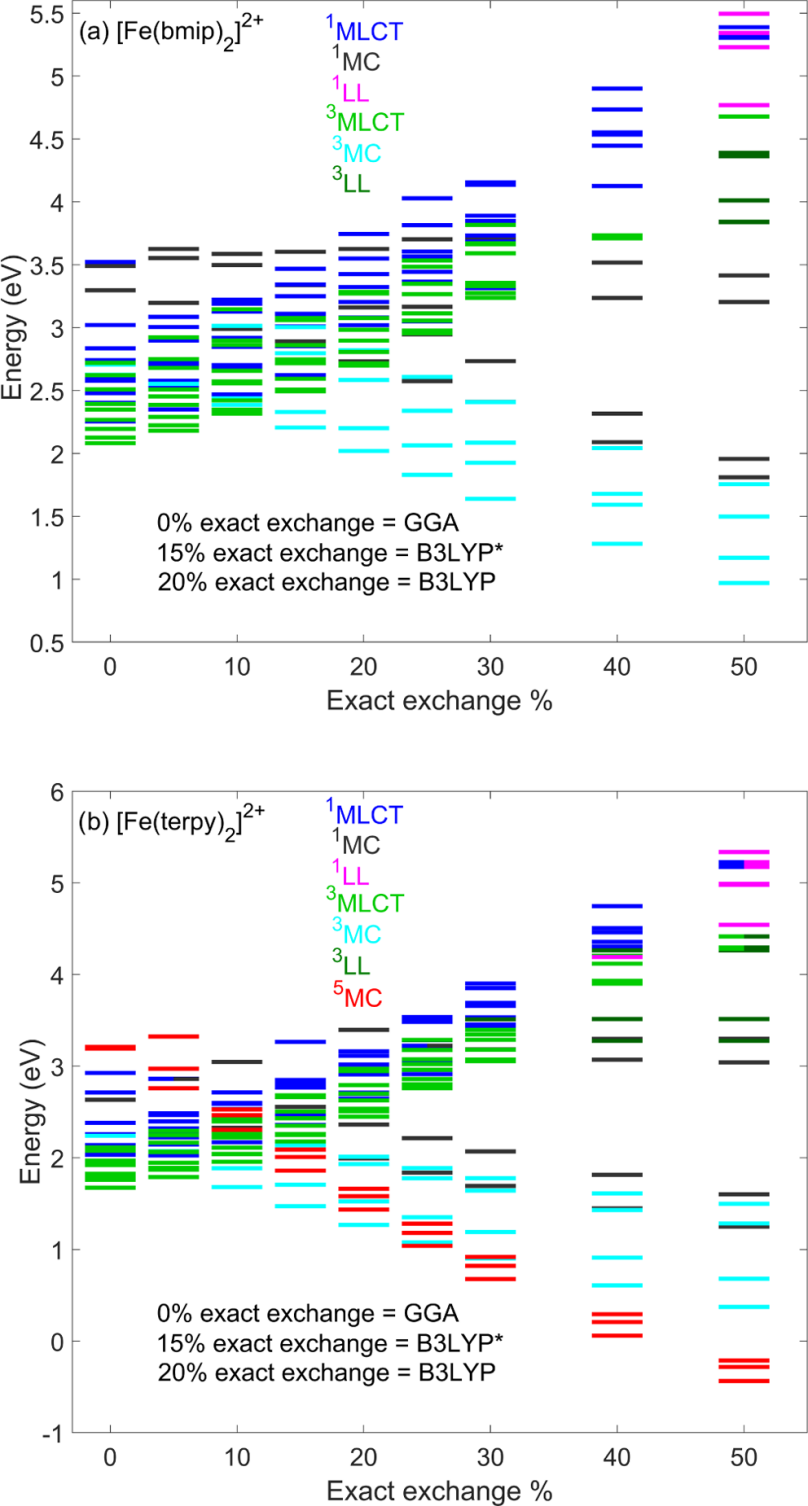
Effect of the fraction of exact exchange on the energetics of (a) [Fe(bmip)_2_]^2+^ and (b) [Fe(terpy)_2_]^2+^ (using the B3LYP functional, calculated at the corresponding ground-state equilibrium geometries). Double-color lines represent excited states with mixed character.

In order to evaluate the effect of exact exchange, which we identified as the main factor affecting the excited-state dynamics, we calculated the energies of the lowest 15 singlet–triplet (and 3 lowest quintets for [Fe(terpy)_2_]^2+^) using the B3LYP DFT/TD-DFT method with varying fractions of exact exchange. The calculations were carried out at the ground-state equilibrium geometries of [Fe(bmip)_2_]^2+^ and [Fe(terpy)_2_]^2+^, optimized by the given B3LYP functional (with varying exact exchange); these results are presented in Figure 10. As seen in the figure, the effect of increasing the exact exchange is opposite for the MLCT and MC states: destabilizing the MLCTs and stabilizing the MCs. Furthermore, within the MC manifold, the stabilizing effect increases with the spin multiplicity (largest for ^5^MC), in agreement with the exchange interaction being largest for the largest spin multiplicity. Interestingly, the energies for 10% exact exchange agree rather well with the corresponding TPSSh energies (see Tables S1–S6 in the Supporting Information), indicating that the decisive factor is indeed the exact exchange (as the fraction of the exact exchange in TPSSh is also 10% but is a meta-hybrid functional). Finally, we note that data obtained using exact exchange above 50% are not shown, as the MC states are clearly overstabilized, and no MLCT states are found within the first lowest 15 singlet and triplet states (^1^LL and ^3^LL states are lowered below the MLCTs as can be seen in Figure 10 for a 50% exact exchange).

**Figure 11:**
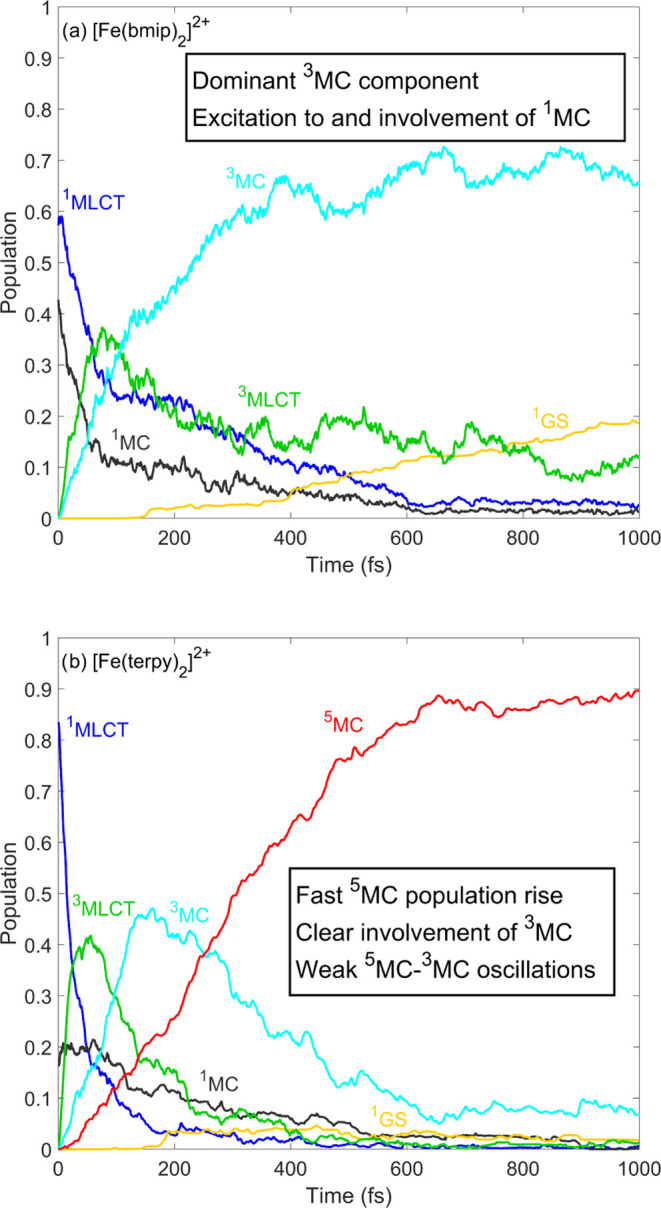
Excited-state population dynamics of (a) [Fe(bmip)_2_]^2+^ and (b) [Fe(terpy)_2_]^2+^ simulated using potentials obtained by optimally tuned LC-BLYP (parameters taken from ref (63) corresponding to “set B”). The shown populations were obtained by averaging over 125 and 106 trajectories for panels (a) and (b), respectively.

The range-separated hybrid functional LC-BLYP deserves a separate discussion. Interestingly, while the population dynamics for [Fe(bmip)_2_]^2+^ simulated by LC-BLYP are very similar as for the other range-separated hybrid CAM-B3LYP and the two global hybrids B3LYP and PBE0, for [Fe(terpy)_2_]^2+^, the population dynamics obtained by LC-BLYP are completely different from those obtained by the other three hybrid functionals. Furthermore, the ^5^MC LVC potential for both complexes is placed energetically significantly higher (by ∼1.5 eV) compared to those calculated by the other three hybrids (see Figures 5 and 9; for ^3^MC states, this energy difference is ca. three times smaller). Interestingly, this LC-BLYP-calculated ^5^MC energy curve for both complexes is very close to those calculated by the GGA methods; it is important to note that this only holds for ^5^MC and not for all of the other singlet and triplet excited states. These observations can be interpreted by noting that (i) the quintet and singlet–triplet excited states in the current methodology are calculated differently by unrestricted DFT (SCF) with imposing the spin multiplicity to be quintet and TD-DFT using the ^1^GS reference (singlets–triplets), and (ii) the part of the LC-BLYP exchange functional that is independent of the range separation in the original formulation is identical to the BLYP exchange functional, which is pure GGA, and exact exchange thus only appears in the variable part. Therefore, the obtained results for LC-BLYP can be explained by the variable (range-separation-dependent) exact exchange acting only effectively in the TD-DFT formulation utilized for the computation of singlet–triplet excited states. Importantly, this leads to an inconsistency in the LC-BLYP-calculated energies of the quintet and singlet–triplet excited states, which is reflected in the qualitatively wrong population dynamics of [Fe(terpy)_2_]^2+^ obtained by the LC-BLYP TSH simulation. It is, however, important to point out that the LC-BLYP functional was employed in its original form. In order to complete our assessment of exchange–correlation functionals, we use the optimally tuned LC-BLYP method from ref (63) (containing 20% exact exchange), for which the best agreement was obtained between TD-DFT and CASPT2 energies, to simulate the excited-stated dynamics of [Fe(bmip)_2_]^2+^ and [Fe(terpy)_2_]^2+^; the results are shown in Figure 11. For both complexes, the results are similar to those obtained by the hybrid functionals such as B3LYP; thus, for [Fe(bmip)_2_]^2+^, the dynamics are dominated by the ^3^MC component with no long-lived ^3^MLCT state, whereas for [Fe(terpy)_2_]^2+^, the fast ^5^MC population rise via clear involvement of ^3^MC states is observed. In fact, for [Fe(terpy)_2_]^2+^, both the nonexponential nature of the ^5^MC population curve and the ^5^MC–^3^MC oscillations are reproduced, albeit the latter are weaker than those identified in the dynamics simulated by B3LYP* (see Figure 6a). Therefore, the LC-BLYP results are drastically improved by optimal tuning, although the performance of B3LYP* and TPSSh is still superior (note, however, that the tuning was not carried out for the studied Fe complexes but for a related [Fe(terpy)_2_]^2+^ derivative).

Finally, we discuss general aspects of our simulations. First, it is important to emphasize that the dynamics occur on full-dimensional potentials; thus, our assessment of the description of excited states by DFT/TD-DFT reflects the exploration of the full nuclear configurational space. However, projection onto a single mode often turns out to be useful, provided that the selected mode is well chosen. This was the case for the principal breathing mode of [Fe(bmip)_2_]^2+^ and [Fe(terpy)_2_]^2+^, which we used to construct one-dimensional potential energy surfaces that were found to be valuable for the interpretation of the differences seen in the dynamics. Second, we recall that all dynamics simulations were carried out on LVC potentials; thus, our assessment is strictly valid only within the LVC approximation. However, the photophysics of [Fe(bmip)_2_]^2+^ and [Fe(terpy)_2_]^2+^ does not involve any large amplitude nuclear motion and the decisive dynamics are expected to occur at geometries only slightly distorted from the equilibrium one, for which the LVC model works rather well; therefore, the utilization of the LVC model is reasonable without causing any significant bias in the obtained results. Lastly, we emphasize that the dynamics are determined by the relative energetics between excited states with various characters and spin multiplicities. We note that these relative energetics cannot be directly extracted from the ground-state absorption spectrum, as the extracted excitation energies are limited to optically bright states, e.g., for low-spin Fe(II) complexes such as [Fe(bmip)_2_]^2+^ and [Fe(terpy)_2_]^2+^, to ^1^MLCT states in the visible region (and dominantly to ^1^LLs in the UV region). Therefore, analysis of the absorption spectrum in itself does not allow assessment of the excited-state energetics discussed in the present work; we demonstrate this aspect in the Supporting Information; see Section S3 and Figure S2. There, we identify the GGA methods (RPBE, OPBE, and BLYP) as those that best reproduce the experimental absorption spectra; it is clear, though, from the present work that GGA DFT/TD-DFT methods do not produce correct excited-state energetics and dynamics (i.e., the MLCT states are overstabilized for both [Fe(bmip)_2_]^2+^ and [Fe(terpy)_2_]^2+^).

## Supporting Information Available

Comparison of B2PLYP, B3LYP*, and TPSSh DFT/TD-DFT energies, comparison of adiabatic B3LYP* and TPSSh potential energy surfaces for [Fe(bmip)_2_]^2+^, TD-DFT-calculated absorption spectra, effect of the Tamm–Dancoff approximation (TDA) on the calculated excitation energies, scalar relativistic effects on the TD-DFT-calculated spin–orbit couplings (SOCs) for [Fe(bmip)_2_]^2+^ (PDF)
LVC parameters (a.u.) and Cartesian coordinates (Å) of the optimized geometries, DFT/TD-DFT SOC matrices for [Fe(bmip)_2_]^2+^ calculated with and without using ZORA (ZIP)
