# Supplementary material for: Simulation of Ultrafast Excited-State Dynamics in Fe(II) Complexes: Assessment of Electronic Structure Descriptions
Source: J Chem Theory Comput. 2025 Jan 3;21(2):560–74. doi: 10.1021/acs.jctc.4c01331 (PMC11780750; doi:10.1021/acs.jctc.4c01331)
Supplement: Supplementary file 1 — ct4c01331_si_001.pdf [file ct4c01331_si_001.pdf]

Supporting Information for "Simulation of Ultrafast Excited-State Dynamics in Fe(II) Complexes: Assessment of Electronic Structure Descriptions"

Mátyás Pápai*

HUN-REN Wigner Research Centre for Physics, P.O. Box 49, H-1525 Budapest, Hungary

Email: papai.matyas@wigner.hun-ren.hu

Contents

S1 Comparison of B2PLYP, B3LYP*, and TPSSh DFT/TD-DFT Energies S2

S2 Comparison of Adiabatic B3LYP* and TPSSh Potential Energy Surfaces for $[Fe(bmip)_2]^{2+}$ S9

S3 TD-DFT Calculated Absorption Spectra S10

S4 Effect of the Tamm-Dancoff Approximation (TDA) on the Calculated Excitation Energies S11

S5 Scalar Relativistic Effects on the TD-DFT Calculated Spin-Orbit Couplings (SOCs) for $[Fe(bmip)_2]^{2+}$ S16

S1 Comparison of B2PLYP, B3LYP*, and TPSSh DFT/TD-DFT Energies

Double hybrid functionals such as B2PLYP offer interesting features such as perturbative doubles correction (D) to TD-DFT. However, such B2PLYP TD-DFT calculations are computationally demanding and analytic TD-DFT gradients are not implemented in the utilized ORCA5.0 software[1–3] (neither in the newest 6.0 version of ORCA). Therefore, we need to limit our assessment of the B2PLYP functional to comparison of vertical excitation energies calculated at the ground-state equilibrium geometry, to those computed by the two best performing DFT/TD-DFT methods, B3LYP* and TPSSh. Note, however, that these vertical excitation energies are those to be found to be most sensitive to the choice of exchange-correlation functional, and are thus mostly responsible for the changes observed in the simulated dynamics.

The results are presented in Tables S1−S6 below, for $[Fe(bmip)_2]^{2+}$ and $[Fe(terpy)_2]^{2+}$. Overall, the agreement between the B2PLYP and B3LYP*/TPSSh energies is rather varying: some of the energies agree rather well, in particular for $[Fe(terpy)_2]^{2+}$, but several MLCT/LL states are overstabilized well below 2 eV. Furthermore, for $[Fe(bmip)_2]^{2+}$, the quintet energies are rather high due to the MP2 correlation favoring the singlet state over the quintet by almost 2 eV (we mention though that no direct experimental reference is available to validate the quintet state of $[Fe(bmip)_2]^{2+}$ as it is not involved in the excited-state dynamics; in fact, a high quintet energy implies that the ^{5}MC state would not be involved in the B2PLYP-simulated dynamics if it were feasible). We note that the appearance of LL excited states is likely a consequence of high exact exchange (53% in B2PLYP), as LL states also appeared for B3LYP with 50% exact exchange (see Figure 10 in the main article). Based on these results and comparison of the B3LYP*/TPSSh dynamics to the experimental ones, the performance of the B2PLYP DFT/TD-DFT method is not expected to reach those of B3LYP* and TPSSh. Furthermore, it is important to emphasize that from a practical point of view, B2PLYP faces important limitations (computational cost, lack of analytic TD-DFT gradients).

The results presented in Tables S1−S6 also offer comparsion of B3LYP* and TPSSh. The overall agreement is acceptable, consistent with variations expected from changing the fraction of exact exchange from 15% (B3LYP*) to 10% (TPSSh). One important aspect is the significant rise of quintet TPSSh energies (∼0.5−0.8 eV) as compared to the B3LYP* values (a consequence of change in the fraction of exact exchange), which certainly shifts the TPSSh-calculated quintet states out of the region at which the excited-state dynamics occur (upon excitation to the lowest-lying optically active ^{1}MLCT state).

Table S1: Comparison of the lowest 15 singlet-singlet vertical excitation energies of $[Fe(bmip)_2]^{2+}$ calculated by B2PLYP, B3LYP*, and TPSSh using the TZVP basis set. All calculations were carried out at the ground-state equilibrium geometry, optimized by the given DFT method. All energies values are given in eV.

State	B2PLYP	State	B3LYP*	State	TPSSh
S_1–^{1}LL	1.607	S_1–^{1}MLCT	2.622	S_1–^{1}MLCT	2.598
S_2–^{1}MLCT/^{1}LL	1.861	S_2–^{1}MLCT	2.622	S_2–^{1}MLCT	2.598
S_3–^{1}MLCT/^{1}LL	1.921	S_3–^{1}MLCT	2.853	S_3–^{1}MLCT	2.861
S_4–^{1}MLCT	2.405	S_4–^{1}MLCT	2.857	S_4–^{1}MLCT	2.867
S_5–^{1}MLCT	2.560	S_5–^{1}MC	2.891	S_5–^{1}MLCT	3.005
S_6–^{1}MLCT	2.560	S_6–^{1}MC	2.891	S_6–^{1}MLCT	3.096
S_7–^{1}MLCT	3.216	S_7–^{1}MLCT	3.009	S_7–^{1}MC	3.190
S_8–^{1}MC	3.473	S_8–^{1}MLCT	3.110	S_8–^{1}MC	3.190
S_9–^{1}MC	3.473	S_9–^{1}MLCT	3.250	S_9–^{1}MLCT	3.328
S_{10}–^{1}LL	3.523	S_{10}–^{1}MLCT	3.250	S_{10}–^{1}MLCT	3.328
S_{11}–^{1}LL	3.523	S_{11}–^{1}MC	3.338	S_{11}–^{1}MLCT	3.352
S_{12}–^{1}LL	3.590	S_{12}–^{1}MLCT	3.343	S_{12}–^{1}MLCT	3.457
S_{13}–^{1}LL	3.590	S_{13}–^{1}MLCT	3.468	S_{13}–^{1}MLCT	3.457
S_{14}–^{1}MLCT	3.977	S_{14}–^{1}MLCT	3.468	S_{14}–^{1}MC	3.648
S_{15}–^{1}MC	4.240	S_{15}–^{1}MC	3.603	S_{15}–^{1}MC	3.815

Table S2: Comparison of the lowest 15 singlet-triplet vertical excitation energies of $[Fe(bmip)_2]^{2+}$ calculated by B2PLYP, B3LYP*, and TPSSh using the TZVP basis set. All calculations were carried out at the ground-state equilibrium geometry, optimized by the given DFT method. All energies values are given in eV.

State	B2PLYP	State	B3LYP*	State	TPSSh
T_1–^{3}MLCT/^{3}LL	1.808	T_1–^{3}MC	2.206	T_1–^{3}MLCT/^{3}MC	2.446
T_2–^{3}LL	1.957	T_2–^{3}MC	2.206	T_2–^{3}MLCT/^{3}MC	2.446
T_3–^{3}MC	2.623	T_3–^{3}MC	2.329	T_3–^{3}MLCT	2.471
T_4–^{3}MC	2.623	T_4–^{3}MLCT	2.495	T_4–^{3}MLCT/^{3}MC	2.486
T_5–^{3}MLCT	2.777	T_5–^{3}MLCT	2.508	T_5–^{3}MLCT/^{3}MC	2.486
T_6–^{3}MLCT	2.777	T_6–^{3}MLCT	2.508	T_6–^{3}MC	2.552
T_7–^{3}MLCT/^{3}LL	2.838	T_7–^{3}MLCT	2.594	T_7–^{3}MLCT	2.560
T_8–^{3}MLCT	3.015	T_8–^{3}MLCT	2.718	T_8–^{3}MLCT	2.689
T_9–^{3}MLCT	3.015	T_9–^{3}MLCT	2.747	T_9–^{3}MLCT	2.754
T_{10}–^{3}MLCT	3.047	T_{10}–^{3}MLCT	2.797	T_{10}–^{3}MLCT	2.852
T_{11}–^{3}MLCT	3.188	T_{11}–^{3}MLCT	2.861	T_{11}–^{3}MLCT	3.016
T_{12}–^{3}MLCT	3.188	T_{12}–^{3}MC	3.004	T_{12}–^{3}MC	3.054
T_{13}–^{3}MC	3.198	T_{13}–^{3}MC	3.004	T_{13}–^{3}MLCT	3.084
T_{14}–^{3}MLCT	3.354	T_{14}–^{3}MLCT	3.061	T_{14}–^{3}MLCT	3.084
T_{15}–^{3}LL	4.240	T_{15}–^{3}MLCT	3.078	T_{15}–^{3}MC	3.331

Table S3: Comparison of the lowest 3 singlet-quintet vertical excitation energies of $[Fe(bmip)_2]^{2+}$ calculated by B2PLYP, B3LYP*, and TPSSh using the TZVP basis set. All calculations were carried out at the ground-state equilibrium geometry, optimized by the given DFT method. All energies values are given in eV.

State	B2PLYP	State	B3LYP*	State	TPSSh
$Q_1-{}^5MC$	4.684	$Q_1-{}^5MC$	3.514	$Q_1-{}^5MC$	3.978
$Q_2-{}^5MC$	4.979	$Q_2-{}^5MC$	3.599	$Q_2-{}^5MC$	4.157
$Q_3-{}^5MC$	4.979	$Q_3-{}^5MC$	3.599	$Q_3-{}^5MC$	4.368

Table S4: Comparison of the lowest 15 singlet-singlet vertical excitation energies of $[Fe(terpy)_2]^{2+}$ calculated by B2PLYP, B3LYP*, and TPSSh using the TZVP basis set. All calculations were carried out at the ground-state equilibrium geometry, optimized by the given DFT method. All energies values are given in eV.

State	B2PLYP	State	B3LYP*	State	TPSSh
$S_1-{}^1$MLCT	1.490	$S_1-{}^1$MC	2.155	$S_1-{}^1$MLCT	2.300
$S_2-{}^1$MLCT	1.728	$S_2-{}^1$MLCT	2.358	$S_2-{}^1$MLCT	2.300
$S_3-{}^1$MLCT	2.080	$S_3-{}^1$MLCT	2.358	$S_3-{}^1$MLCT	2.388
$S_4-{}^1$MLCT	2.117	$S_4-{}^1$MLCT	2.442	$S_4-{}^1$MLCT	2.417
$S_5-{}^1$MC	2.299	$S_5-{}^1$MLCT	2.464	$S_5-{}^1$MC	2.577
$S_6-{}^1$MLCT	2.625	$S_6-{}^1$MC	2.550	$S_6-{}^1$MLCT	2.586
$S_7-{}^1$MLCT	2.625	$S_7-{}^1$MC	2.550	$S_7-{}^1$MLCT	2.589
$S_8-{}^1$MLCT/^{1}LL	2.653	$S_8-{}^1$MLCT	2.665	$S_8-{}^1$MLCT	2.695
$S_9-{}^1$MC	2.689	$S_9-{}^1$MLCT	2.673	$S_9-{}^1$MLCT	2.695
$S_{10}-{}^1$MC	2.689	$S_{10}-{}^1$MLCT	2.766	$S_{10}-{}^1$MLCT	2.738
$S_{11}-{}^1$MLCT	2.839	$S_{11}-{}^1$MLCT	2.766	$S_{11}-{}^1$MLCT	2.759
$S_{12}-{}^1$MLCT	2.839	$S_{12}-{}^1$MLCT	2.809	$S_{12}-{}^1$MLCT	2.759
$S_{13}-{}^1$LL	2.851	$S_{13}-{}^1$MLCT	2.848	$S_{13}-{}^1$MC	2.956
$S_{14}-{}^1$LL	2.901	$S_{14}-{}^1$MLCT	2.848	$S_{14}-{}^1$MC	2.956
$S_{15}-{}^1$LL	2.901	$S_{15}-{}^1$MLCT	3.263	$S_{15}-{}^1$MLCT	3.209

Table S5: Comparison of the lowest 15 singlet-triplet vertical excitation energies of $[Fe(terpy)_2]^{2+}$ calculated by B2PLYP, B3LYP*, and TPSSh using the TZVP basis set. All calculations were carried out at the ground-state equilibrium geometry, optimized by the given DFT method. All energies values are given in eV.

State	B2PLYP	State	B3LYP*	State	TPSSh
T_1–^{3}MC	1.411	T_1–^{3}MC	1.471	T_1–^{3}MC	1.790
T_2–^{3}MC	1.761	T_2–^{3}MC	1.699	T_2–^{3}MC	2.022
T_3–^{3}MC	1.761	T_3–^{3}MC	1.699	T_3–^{3}MC	2.022
T_4–^{3}MC	2.264	T_4–^{3}MC	2.081	T_4–^{3}MLCT	2.082
T_5–^{3}MC	2.264	T_5–^{3}MC	2.081	T_5–^{3}MLCT	2.151
T_6–^{3}MC	2.383	T_6–^{3}MC	2.136	T_6–^{3}MLCT	2.162
T_7–^{3}MLCT	2.884	T_7–^{3}MLCT	2.177	T_7–^{3}MLCT	2.162
T_8–^{3}MLCT	2.975	T_8–^{3}MLCT	2.248	T_8–^{3}MLCT	2.225
T_9–^{3}LL	3.230	T_9–^{3}MLCT	2.261	T_9–^{3}MLCT	2.373
T_{10}–^{3}LL	3.243	T_{10}–^{3}MLCT	2.261	T_{10}–^{3}MLCT	2.377
T_{11}–^{3}MLCT	3.333	T_{11}–^{3}MLCT	2.349	T_{11}–^{3}MC	2.397
T_{12}–^{3}MLCT	3.333	T_{12}–^{3}MLCT	2.430	T_{12}–^{3}MC	2.501
T_{13}–^{3}LL	3.395	T_{13}–^{3}MLCT	2.508	T_{13}–^{3}MC	2.501
T_{14}–^{3}LL	3.395	T_{14}–^{3}MLCT	2.665	T_{14}–^{3}MLCT	2.539
T_{15}–^{3}LL	3.590	T_{15}–^{3}MLCT	2.682	T_{15}–^{3}MLCT	2.602

Table S6: Comparison of the lowest 3 singlet-quintet vertical excitation energies of $[Fe(terpy)_2]^{2+}$ calculated by B2PLYP, B3LYP*, and TPSSh using the TZVP basis set. All calculations were carried out at the ground-state equilibrium geometry, optimized by the given DFT method. All energies values are given in eV.

State	B2PLYP	State	B3LYP*	State	TPSSh
$Q_1-{}^5MC$	1.838	$Q_1-{}^5MC$	1.927	$Q_1-{}^5MC$	2.351
$Q_2-{}^5MC$	1.942	$Q_2-{}^5MC$	1.935	$Q_2-{}^5MC$	2.643
$Q_3-{}^5MC$	2.080	$Q_3-{}^5MC$	1.935	$Q_3-{}^5MC$	2.728

S2 Comparison of Adiabatic B3LYP* and TPSSh Potential Energy Surfaces for $[Fe(bmip)_2]^{2+}$

As mentioned in the main article and seen in Table S2, several of the lowest TPSSh-calculated triplet states of $[Fe(bmip)_2]^{2+}$at the ground-state equilibrium geometry possess mixed ^{3}MLCT/^{3}MC character. This indicates ^{3}MLCT/^{3}MC branching (which is observed experimentally), but at the same time, it does not allow access to the population dynamics (as the separation of ^{3}MLCT and ^{3}MC populations is not possible). Therefore, for the assessment of the TPSSh DFT/TD-DFT method for $[Fe(bmip)_2]^{2+}$, we follow a different approach by comparing the adiabatic TPSSh and B3LYP* potential energy surfaces along the breathing mode (obtained by performing DFT/TD-DFT calculations ad the ground-state equilibrium geometry and geometries displaced along the breathing mode). We note that the reason for choosing the adiabatic electronic basis is that certain diabatic TPSSh triplet potentials would have mixed ^{3}MLCT/^{3}MC character, in contrast to the pure character of all B3LYP* potentials. Furthermore, we mention that the B3LYP* DFT/TD-DFT method is chosen as a reference due to its reliability observed in the main article by comparison of the simulated population dynamics to those extracted experimentally by time-resolved X-ray emission spectroscopy (XES).

The obtained B3LYP* and TPSSh DFT/TD-DFT poential energy surfaces are shown in Figure S1. We observe a rather good agreement with the only apparent difference being the modulation of the ^{3}MLCT/^{3}MC energetics of the lowest-triplet states. These results suggest similar dynamics for B3LYP* and TPSSh, with some possible differences in the ^{3}MLCT/^{3}MC branching ratio in favor of the ^{3}MLCT component for TPSSh (as the lower fraction of exact exchange lowers the MLCTs and raises the MCs).

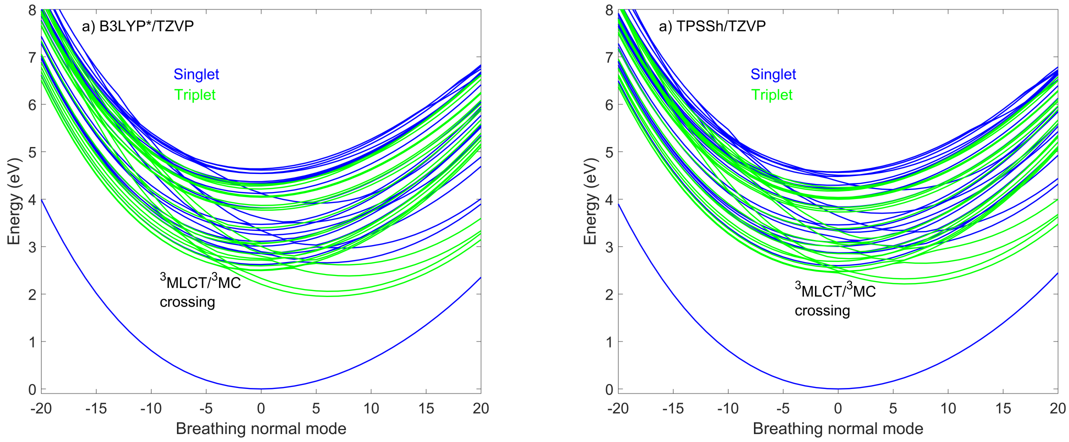

Figure S1: Comparison of adiabatic a) B3LYP* and b) TPSSh DFT/TD-DFT potential energy surfaces of $[Fe(bmip)_2]^{2+}$ along the breathing normal mode. Singlet and triplet surfaces are shown in blue and green, respectively.

S3 TD-DFT Calculated Absorption Spectra

The conventional methodology for assessing the reliability of excited-state calculations is based on the comparison of experimental and calculated absorption spectra. However, this approach is limited to probe excited states that are optically active (i.e., with considerable transition dipole moment for an electronic transition originating from the ground state). In the visible region for low-spin Fe(II) complexes such as $[Fe(bmip)_2]^{2+}$ and $[Fe(terpy)_2]^{2+}$, probed states are limited to ^{1}MLCTs, as transition dipole moments for MC states are small (in fact, zero for complexes with perfect octahedral symmetry due to the Laporte selection rule) and singlet-triplet spin-orbit coupling (SOC) is weak enough such that contribution from triplet states is practically negligible. This means that the visible absorption spectrum only contains information about ^{1}MLCT states (and ^{1}LL states and possibly ^{1}MLCTs/^{1}LMCTs in the UV region), as opposed to the relative energetics between states with different electronic character and spin multiplicity, which determine the excited-state dynamics. Furthermore, the absorption spectrum is taken in equilibrium of the ground-state, while the full-dimensional excited-state dynamics explore an extended region of the potential energy surface.

It is thus insightful to assess the performance of the utilized exchange-correlation functionals by calculation of the absorption spectrum for the electronic ground state. Figure S2 displays the TD-DFT calculated spectra for $[Fe(bmip)_2]^{2+}$ and $[Fe(terpy)_2]^{2+}$ (50 singlet states were calculated by TD-DFT at the corresponding ground-state equilibrium geometry; a pseudo-Voigt profile was applied to take into account the spectral broadening, with 1000 cm^{-1} FWHM for both the Gaussian and Lorentzian components). It is clear from the figure that the observed absorption peak (with ^{1}MLCT character) is located at higher wavelengths (lower energy) for the GGAs and lower wavelengths (higher energy) as the fraction of exact exchange increases; this is in agreement with Figure 10 of the main article (the MLCT energies increase with increasing exact exchange). Furthermore, we find that all TD-DFT calculated peaks are located at lower wavelength as compared to the experimental absorption peaks, with the GGA-calculated peaks (RPBE, OPBE, BLYP) reproducing best the experimental peaks position (the experimental data is taken from ref. 4). This is in stark contrast to our dynamics results, which clearly show that GGA DFT/TD-DFT methods overstabilize the MLCT states. These results demonstrate that the relative MLCT-MC energetics are those that govern the excited-state dynamics, which might be rather different for a given method than the assessment based on inspection of the absorption spectrum. We note that for $[Fe(bmip)_2]^{2+}$, the second MLCT peak observed in the experiment at ca. 390 nm is only resolved for the GGA methods (albeit at lower wavelength than the experimental one), as for the other functionals including exact exchange this band seemingly gets merged with the intense band in the UV region. Moreover, B2PLYP yields a small peak around 650 nm that is an outlier regarding comparison to all other calculated spectra as well as the experimental one.

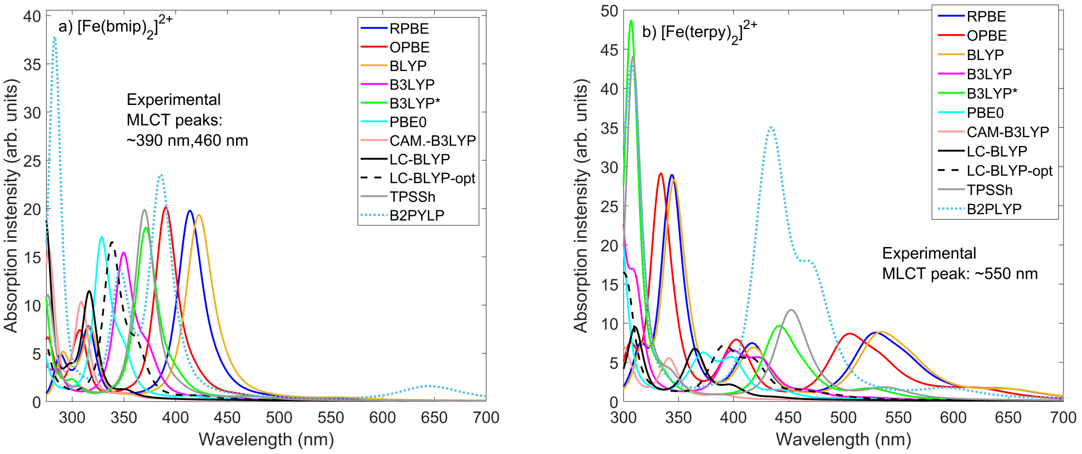

Figure S2: TD-DFT calculated absorption spectrum for a) $[Fe(bmip)_2]^{2+}$ and b) $[Fe(terpy)_2]^{2+}$. Obtained by calculating the lowest 50 TD-DFT singlet excited states at the corresponding ground-state equilibrium geometry. The experimental data is taken from ref. 4.

S4 Effect of the Tamm-Dancoff Approximation (TDA) on the Calculated Excitation Energies

The Tamm-Dancoff approximation (TDA) is an approach widely used in TD-DFT as it improves the energetics;[5,6] it was also found that it can improve non-adiabatic couplings.[5] As a part of the present work, we have evaluated the effect of TDA on the 15 lowest B3LYP* TD-DFT singlet-singlet and singlet-triplet vertical excitation energies of $[Fe(bmip)_2]^{2+}$ and $[Fe(terpy)_2]^{2+}$(computed at the corresponding ground-state equilibrium geometries); the result are shown in Tables S7−S10. The most important conclusion obtained by the analysis of this data is that the utilization of the TDA does not have any considerable effect on the MLCT-MC energetics. The TDA-calculated excitation energies are systematically slightly higher than those calculated by full TD-DFT with a largest difference being ∼0.2 eV (however, most of the differences are significantly smaller, in particular for the singlet states).

Table S7: Comparison of TDA/TD-DFT and full TD-DFT (without using TDA) B3LYP* singlet-singlet excitation energies of $[Fe(bmip)_2]^{2+}$. All calculations were carried out at the ground-state equilibrium geometry, optimized by B3LYP*/TZVP. All energies values are given in eV.

State	TDA/TD-DFT	State	Full TD-DFT
S_1–^{1}MLCT	2.622	S_1–^{1}MLCT	2.610
S_2–^{1}MLCT	2.622	S_2–^{1}MLCT	2.610
S_3–^{1}MLCT	2.853	S_3–^{1}MLCT	2.846
S_4–^{1}MLCT	2.857	S_4–^{1}MLCT	2.851
S_5–^{1}MC	2.891	S_5–^{1}MC	2.873
S_6–^{1}MC	2.891	S_6–^{1}MC	2.873
S_7–^{1}MLCT	3.009	S_7–^{1}MLCT	2.992
S_8–^{1}MLCT	3.110	S_8–^{1}MLCT	3.083
S_9–^{1}MLCT	3.250	S_9–^{1}MLCT	3.235
S_{10}–^{1}MLCT	3.250	S_{10}–^{1}MLCT	3.235
S_{11}–^{1}MC	3.338	S_{11}–^{1}MLCT	3.286
S_{12}–^{1}MLCT	3.343	S_{12}–^{1}MC	3.320
S_{13}–^{1}MLCT	3.468	S_{13}–^{1}MLCT	3.460
S_{14}–^{1}MLCT	3.468	S_{14}–^{1}MLCT	3.460
S_{15}–^{1}MC	3.603	S_{15}–^{1}MC	3.501

Table S8: Comparison of TDA/TD-DFT and full TD-DFT (without using TDA) B3LYP* singlet-triplet excitation energies of $[Fe(bmip)_2]^{2+}$. All calculations were carried out at the ground-state equilibrium geometry, optimized by B3LYP*/TZVP. All energies values are given in eV.

State	TDA/TD-DFT	State	Full TD-DFT
T_1–^{3}MC	2.206	T_1–^{3}MC	2.082
T_2–^{3}MC	2.206	T_2–^{3}MC	2.082
T_3–^{3}MC	2.329	T_3–^{3}MC	2.227
T_4–^{3}MLCT	2.495	T_4–^{3}MLCT	2.385
T_5–^{3}MLCT	2.508	T_5–^{3}MLCT	2.479
T_6–^{3}MLCT	2.508	T_6–^{3}MLCT	2.479
T_7–^{3}MLCT	2.594	T_7–^{3}MLCT	2.523
T_8–^{3}MLCT	2.718	T_8–^{3}MLCT	2.644
T_9–^{3}MLCT	2.747	T_9–^{3}MC	2.693
T_{10}–^{3}MLCT	2.797	T_{10}–^{3}MLCT	2.725
T_{11}–^{3}MLCT	2.861	T_{11}–^{3}MLCT	2.846
T_{12}–^{3}MC	3.004	T_{12}–^{3}MC	2.919
T_{13}–^{3}MC	3.004	T_{13}–^{3}MC	2.919
T_{14}–^{3}MLCT	3.061	T_{14}–^{3}MLCT	3.027
T_{15}–^{3}MLCT	3.078	T_{15}–^{3}MLCT	3.055

Table S9: Comparison of TDA/TD-DFT and full TD-DFT (without using TDA) B3LYP* singlet-singlet excitation energies of $[Fe(terpy)_2]^{2+}$. All calculations were carried out at the ground-state equilibrium geometry, optimized by B3LYP*/TZVP. All energies values are given in eV.

State	TDA/TD-DFT	State	Full TD-DFT
S_1–^{1}MC	2.155	S_1–^{1}MC	2.144
S_2–^{1}MLCT	2.358	S_2–^{1}MLCT	2.346
S_3–^{1}MLCT	2.358	S_3–^{1}MLCT	2.346
S_4–^{1}MLCT	2.442	S_4–^{1}MLCT	2.436
S_5–^{1}MLCT	2.464	S_5–^{1}MLCT	2.458
S_6–^{1}MC	2.550	S_6–^{1}MC	2.532
S_7–^{1}MC	2.550	S_7–^{1}MC	2.532
S_8–^{1}MLCT	2.665	S_8–^{1}MLCT	2.640
S_9–^{1}MLCT	2.673	S_9–^{1}MLCT	2.656
S_{10}–^{1}MLCT	2.766	S_{10}–^{1}MLCT	2.756
S_{11}–^{1}MLCT	2.766	S_{11}–^{1}MLCT	2.756
S_{12}–^{1}MLCT	2.809	S_{12}–^{1}MLCT	2.785
S_{13}–^{1}MLCT	2.848	S_{13}–^{1}MLCT	2.839
S_{14}–^{1}MLCT	2.848	S_{14}–^{1}MLCT	2.839
S_{15}–^{1}MLCT	3.263	S_{15}–^{1}MLCT	3.119

Table S10: Comparison of TDA/TD-DFT and full TD-DFT (without using TDA) B3LYP* singlet-triplet excitation energies of $[Fe(terpy)_2]^{2+}$. All calculations were carried out at the ground-state equilibrium geometry, optimized by B3LYP*/TZVP. All energies values are given in eV.

State	TDA/TD-DFT	State	Full TD-DFT
T_1–^{3}MC	1.471	T_1–^{3}MC	1.268
T_2–^{3}MC	1.699	T_2–^{3}MC	1.537
T_3–^{3}MC	1.699	T_3–^{3}MC	1.537
T_4–^{3}MC	2.081	T_4–^{3}MC	1.948
T_5–^{3}MC	2.081	T_5–^{3}MC	1.948
T_6–^{3}MC	2.136	T_6–^{3}MC	2.017
T_7–^{3}MLCT	2.177	T_7–^{3}MLCT	2.100
T_8–^{3}MLCT	2.248	T_8–^{3}MLCT	2.192
T_9–^{3}MLCT	2.261	T_9–^{3}MLCT	2.234
T_{10}–^{3}MLCT	2.261	T_{10}–^{3}MLCT	2.234
T_{11}–^{3}MLCT	2.349	T_{11}–^{3}MLCT	2.331
T_{12}–^{3}MLCT	2.430	T_{12}–^{3}MLCT	2.424
T_{13}–^{3}MLCT	2.508	T_{13}–^{3}MLCT	2.448
T_{14}–^{3}MLCT	2.665	T_{14}–^{3}MLCT	2.632
T_{15}–^{3}MLCT	2.682	T_{15}–^{3}MLCT	2.670

S5 Scalar Relativistic Effects on the TD-DFT Calculated Spin-Orbit Couplings (SOCs) for $[Fe(bmip)_2]^{2+}$

In ref. 7, we calculated the TD-DFT SOCs of $[Fe(bmip)_2]^{2+}$using B3LYP*/TZVP, without accounting for scalar relativistic effects. As part of the present work, we assessed the effect of using the zeroth-order regular approximation (ZORA)[8,9] and the ZORA-TZVP basis in the calculation of the SOCs between the 20 lowest singlet and triplet excited states. The calculated SOC matrices (we have taken the absolute value of the complex numbers; all SOCs are given in a.u.) are provided in the supplementary data files "Fe-CAB-ABSSOC-WITH_ZORA.dat" and "Fe-CAB-ABSSOC-WITHOUT_ZORA.dat" (presenting 80*80 SOC matrices in the present pdf file is not practical). Analysis of these SOC matrices reveal rather good agreement between SOCs calculated with and without using ZORA (in several cases, only few cm^{-1} differences are observed), indicating that the application of ZORA would not have any significant effect on the dynamics through the SOCs. We note that the state indices may differ in some cases for the TD-DFT calculations with and without ZORA (due to changes in state ordering and/or degenerate components).

References

[1] Neese, F. *WIREs Comput. Mol. Sci.* **2012**, *2*, 73–78.

[2] Neese, F.; Wennmohs, F.; Becker, U.; Riplinger, C. *J. Chem. Phys.* **2020**, *152*, 224108.

[3] Neese, F. *Wiley Interdiscip. Rev. Comput. Mol. Sci.* **2022**, *12*, e1606.

[4] Liu, Y.; Harlang, T.; Canton, S. E.; Chábera, P.; Suárez-Alcántara, K.; Fleckhaus, A.; Vithanage, D. A.; Göransson, E.; Corani, A.; Lomoth, R.; Sundström, V.; Wärnmark, K. *Chem. Commun.* **2013**, *49*, 6412–6414.

[5] Hu, C.; Sugino, O.; Watanabe, K. *J. Chem. Phys.* **2014**, *140*, 054106.

[6] Liang, J.; Feng, X.; Hait, D.; Head-Gordon, M. *J. Chem. Theory Comput.* **2022**, *18*, 3460–3473.

[7] Pápai, M.; Rozgonyi, T.; Vankó, G. *J. Mater. Chem. A* **2023**, *11*, 25955–25962.

[8] Lenthe, E. v.; Baerends, E. J.; Snijders, J. G. *J. Chem. Phys.* **1993**, *99*, 4597–4610.

[9] van Wüllen, C. *J. Chem. Phys.* **1998**, *109*, 392–399.
